# *In Silico* Perspective on Avobenzone, Octisalate, Octocrylene, Homosalate, and Bemotrizinol as Organic UV Filters Using DFT, TD-DFT, and Molecular Dynamics

**DOI:** 10.1021/acsomega.5c09234

**Published:** 2026-02-02

**Authors:** Maria E. Rigoni, Sergio R. de Lazaro, Lucas Stori de Lara

**Affiliations:** † Department of Chemistry, 67883 State University of Ponta Grossa, Av. Carlos Cavalcanti, Uvaranas, Ponta Grossa 4748, Brazil; ‡ Department of Physics, 74362 State University of Ponta Grossa, Av. Carlos Cavalcanti, Uvaranas, Ponta Grossa 4748, Brazil

## Abstract

Ultraviolet radiation is the leading cause of skin damage, such as burns, premature aging, and the development of skin cancer. Organic sunscreens are widely used in the cosmetics industry due to their ability to absorb ultraviolet radiation and dissipate it as heat. These compounds can be susceptible to photochemical instability, which compromises their effectiveness over time and also poses environmental risks, particularly in aquatic ecosystems. This study investigated the electronic properties, absorption spectra, and solvent effects of five widely used organic UV filters: Avobenzone, Homosalate, Octisalate, Octocrylene, and Bemotrizinol. The analyses were performed through DFT and TD-DFT calculations. Solvent effects were evaluated using an implicit solvent model and complemented by molecular dynamics simulations in an aqueous environment.

## Graphical abstract

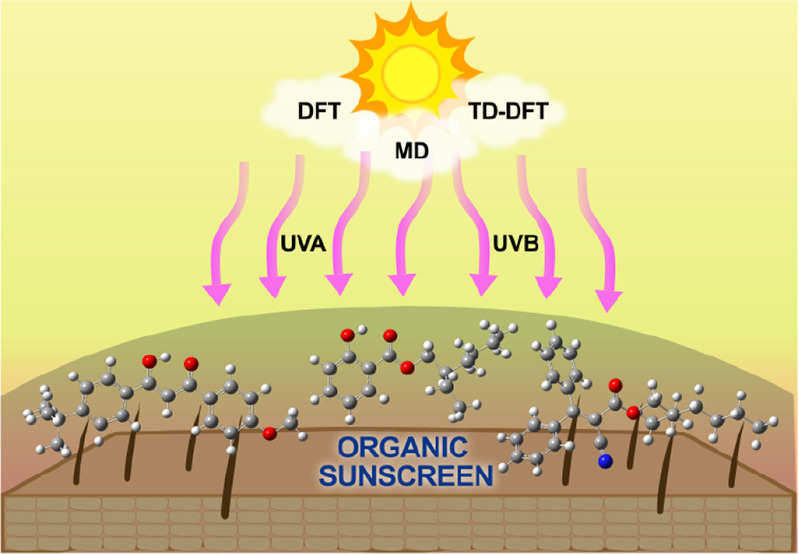

## Introduction

The spectral range that reaches Earth’s surface primarily consists of ultraviolet (UV, 100–400 nm), visible (Vis, 400–700 nm), and infrared (IR, >700 nm) radiations. UV radiation can cause immediate or cumulative damage to the body due to its high energy and short wavelength. It is further divided into UV-type A (UVA, 315–400 nm), UV-type B (UVB, 280–315 nm), and UV-type C (UVC, 100–280 nm). The ozone layer acts as a natural chemical-physical filter. It intercepts the most harmful UVC radiation, allowing life on Earth. Additionally, it significantly absorbs UVB radiation, reducing its intensity at the surface and thereby mitigating its biological effects.^1–5^

The biological effects include erythemas, burns, premature aging, and skin cancer in more severe cases.^6,7^ Skin cancer is one of the most common diseases and is categorized into two types: melanoma and nonmelanoma (carcinomas).^8^ Given these considerations, it is crucial to adopt measures that minimize the harmful effects of the excessive UV radiation. An effective way to reduce exposure to high levels of UV radiation is through the use of sunscreens (photoprotectors), which act as barriers against UVA and UVB radiation. The cosmetics industry widely uses chemical filters based on organic molecules that are capable of absorbing ultraviolet radiation and dissipating the absorbed energy as heat. These organic compounds are mostly used in conjunction with inorganic filters, such as TiO_2_ and ZnO, in order to broaden the absorption range and improve the stability of these formulations.^9–11^

The performance of sunscreens is influenced by various factors, including the photochemical stability of UV filters, their degradation potential, toxicity, skin absorption capacity, solubility in aqueous media, and others.^12–14^ Despite the proven effectiveness of organic filters, the photostability of some compounds can change over time due to different photochemical processes. Avobenzone, for example, exists in two tautomeric forms, keto and enol, and undergoes photoisomerization between these structures upon exposure to UV radiation. While the enol form is responsible for the desired UVA absorption, the keto form is more reactive and susceptible to photodegradation, which can lead to the formation of free radicals and a significant loss in absorption efficiency.^14–16^ Due to its photoinstability, avobenzone can be stabilized by certain UV filters, such as Bemotrizinol, which helps mitigate these effects.^17^

Furthermore, in recent years, studies have indicated the presence of organic filters in aquatic environments, which are connected to the toxicity of marine organisms. Effects such as coral bleaching,^18^ endocrine alterations in fish,^19^ and accumulation in filter organisms^20^ have motivated the development of safer, biodegradable, and sustainable alternatives.^21^ In addition to the environmental impact, there are also concerns regarding toxicity to human health.^22,23^ Regulatory agencies establish maximum allowable concentrations for UV filters to protect human health. Organizations such as the U.S. Food and Drug Administration (FDA), the European Commission (EU Cosmetics Regulation), and the Scientific Committee on Consumer Safety (SCCS) define these limits primarily based on toxicological assessments.

Given this context, researchers have used the TD-DFT methodology to investigate and describe the behavior of electronic excitations in molecular systems, including compounds such as UV filters.^21,24^ In this methodology, the energy of the excited state of the molecule *E*_vert‑abso_ is determined by the difference between the energies of the excited *E*_ES_(*R*_GS_) and ground states *E*_GS_(*R*_GS_), both calculated in the optimized geometry of the ground state.^25,26^

1 $$E_{\mathrm{vert}‐\mathrm{abso}}=E_{\mathrm{ES}}\left(R_{\mathrm{GS}}\right)-E_{\mathrm{GS}}\left(R_{\mathrm{GS}}\right)$$

This representation is known as adiabatic excitation and promotes a change in the electronic state without any geometric relaxation of the structure; i.e., the transitions occur without altering the nuclear positions of the geometry in the ground state.

Moreover, studies have shown that the solvent effect has a significant influence on the optical properties of organic compounds, including UV filters.^27^ A bathochromic effect (red shift) is observed in the absorption spectra as the solvent polarity increases, indicating greater electronic stability. This effect is even more pronounced in protic solvents due to the formation of hydrogen bonds.^28–31^ Although Avobenzone has been extensively investigated through DFT and TD-DFT approaches regarding its tautomers, solvent effects, and photodegradation mechanisms, most other UV filters have not been studied to the same extent. For example, reported theoretical absorption values for Octocrylene often differ from experimental results, revealing an incomplete understanding of its behavior.^32^ Avobenzone remains in this study because it is widely used, and its keto and enol forms show distinct absorption profiles, providing a benchmark for evaluating computational methods on other molecules. This combined approach helps check how well the functionals and basis sets used here perform compared with those already reported in the literature or that could be used in future studies. Doing so makes it easier to predict which molecules might show promising absorption properties and helps focus experimental work on the most likely candidates.

The 70th Session of the United Nations (UN) General Assembly approved Resolutions 65/1 and 69/244, as well as Decision 69/557, which established the post-2015 development agenda with 17 Sustainable Development Goals (SDGs). Thus, SDG 3 (Good Health and Well-being), SDG 12 (Responsible Consumption and Production), SDG 14 (Life Below Water), and SDG 15 (Life on Land) were followed.

The present study employed a TD-DFT/B3LYP and molecular dynamics framework to simulate the molecular behavior of organic UV filters, including Avobenzone, Octocrylene, Octisalate, Homosalate, and Bemotrizinol. The analyzed properties included vertical excitations in singlet and excited singlet states, absorption spectra, frontier orbitals, and solvent effects in water, methanol, and ethanol. The molecular dynamics simulations simulated the molecular aggregation in the aqueous phase.

## Materials and Methods

### Quantum Level Approach

Butyl methoxydibenzoylmethane (Avobenzone), homomenthyl salicylate (Homosalate), ethylhexyl salicylate (Octisalate), octocrylene, and bis-ethylhexyl-oxyphenol methoxyphenyl triazine (Bemotrizinol) were selected for this study. Density Functional Theory (DFT)^33,34^ using the Becke three-parameter functional (B3LYP)^35–37^ with the 6-31+G­(d)^38,39^ basis set simulated molecular geometries in the singlet ground state through complete molecular relaxation. The thermochemical conditions imposed were a vacuum, absolute zero temperature, a gaseous state, no interacting field, and a unimolecular quantity. The convergence criterion adopted for the self-consistent field (SCF) calculation was 10^–8^ a.u., while the geometry optimization was 10^–8^ au with both ″tight″ and ″very tight″ options. Posteriorly, the vibrational simulations tested the atomic positions of the relaxed geometries, confirming the energy minima in the absence of imaginary frequencies.

After obtaining the ground-state geometries, the time dependent-DFT (TD-DFT)^40–44^ with B3LYP approach calculations were performed under the same conditions to develop the excited singlet states of the molecules, evaluating a total of 10 states. Absorption spectra, vertical excitation energies, and excitation path contributions were analyzed.

The Polarizable Continuum Method (PCM),^45^ which employs the implicit solvation approach, accounts for the solvent’s influence on the molecules. The solvent effect is treated as a continuous medium with the dielectric constant *ε*. The solute–solvent interaction occurred with water (ϵ = 78.355), methanol (ϵ = 32.613), and ethanol (ϵ = 24.852), polar protic solvents. The fundamental geometries were relaxed under each solvent effect. Again, the new geometries were subjected to vibrational frequencies and excited-state calculations using the same framework as that in the gas phase. The Gaussian09 ^46^ software was used to calculate the molecular geometries, and the GaussView 6 ^47^ program was then used to analyze these geometries.

### Molecular Dynamics Simulations

The Large Atomic/Molecular Massively Parallel Simulator (LAMMPS)^48^ program performed classical molecular dynamics. The simulated molecular systems included Avobenzone (keto and enol), Bemotrizinol, Octisalate A, Octocrylene, and Homosalate B. The parameters for the Consistent Valence Force Field (CVFF)^49^ were the Mulliken’s partial charges calculated from the DFT approach. The description of the solvent was achieved by simulating water molecules using the Extended Simple Point Charge (SPCE) and the force field developed for simulating HF (SPCE-FH)^50^ model. The employed force field incorporates a three-point charge model that explicitly includes van der Waals and Coulomb interactions. The system’s internal flexibility is addressed by including internal degrees of freedom, which are modeled via harmonic terms for the O–H bond stretching and the H–O–H angle bending.

In the first stage, the calculations consider each system individually to ensure the thermodynamic equilibrium properties. More details are described in the Supporting Information, specifically Table S2, Figures S9, and S10. Next, models of boxes with dimensions *L*_*x*_ × *L*_*y*_ × *L*_*z*_, where *L*_*x*_ = *L*_*y*_ = 10.0 nm and *L*_*z*_ = 5.0 nm, containing each of the molecules in water, were created to form the final systems. Then, the equilibrium phase for each one of the fluids was obtained through a sequence of calculations in *NVE* (microcanonical ensemble), *NVT* (canonical ensemble), and *NPT* (isothermal–isobaric ensemble) at a pressure of 1 atm and a temperature of 300 K. The times of 1, 50, and 20 ps were used for the *NVE*, *NVT*, and *NPT* ensemble calculations, respectively. Furthermore, the Nose–Hoover^51^ thermostat controlled the temperature, and the Andersen barostat^52^ controlled the pressure. Complementary physical descriptions included the periodic boundary condition and long-range electrostatic interactions, which were handled through the Particle–Particle–Particle-Mesh (PPPM) method^53^ in reciprocal space. The time step for the integration was 0.5 fs. In all calculations, van der Waals interactions applied a cutoff radius of 10 Å. After the equilibrium phase, the density profiles were calculated over a 40 ns time interval. The 20 ns equilibration interval determined the simulations, while a 40 ns time period defined the production phase for density profiles, radial distribution functions, and the radius of gyration.

For the density profiles, we consider the *x*-plane of the computational box and take small, perpendicular slices along the *z*-axis. The density as a function of *z* is then assumed by conceptually dividing the physical system into *N* rectangular slabs with a thickness Δ*z* of 0.1 Å. The *N*_*zi*_ is the number of molecules or atoms in the *i*-th layer after an integration interval *j*, and *M* is the mass of each molecule or atom. The density after a specific time interval is given by

2 $$\rho \left(x_{i}\right)=\left\langle \rho \right\rangle =\left\langle \frac{N_{zi}M}{L_{x}L_{y}\Delta z}\right\rangle$$

In general, we define this equation as a density such that *L*_*x*_ and *L*_*y*_ are the lengths. As for the volumetric profiles, the temporal averages of the atomic positions of the solute particles are considered as a function of the box volume.

## Results and Discussion

### Molecular Geometry

The molecular geometries relaxation at the B3LYP/6-31+G­(d) quantum level in the singlet ground state and vacuum showed the absence of imaginary frequencies, resulting in stable structures (Figure 1). Considering the salicylate group in the Octisalate and Homosalate molecules, there is the A conformation, indicated by the coupling between hydrogen H(1) of the hydroxyl group and the carbonylic oxygen O(10), while the B conformation is between the hydrogen H(1) uncoupled with the carbonyl oxygen O(11). The A compounds exhibited planarity in the salicylate group, with dihedral angles close to 0° or 180°, regardless of the hydroxyl orientation. The A Octisalate and A Homosalate conformations are planar with −0.43 and 0.06° in dihedral angle (Figure 1), due to the strong intramolecular interaction of the hydrogen bond, characterized by smaller distances of 1.745 and 1.739 Å between the hydrogen H(1) and oxygen atoms O(10) and O(11), respectively. The influence of hydrogen bonding on the molecular geometry, as well as its contribution to molecular stability, is consistent with other previous studies.^54–56^

**Figure 1:**
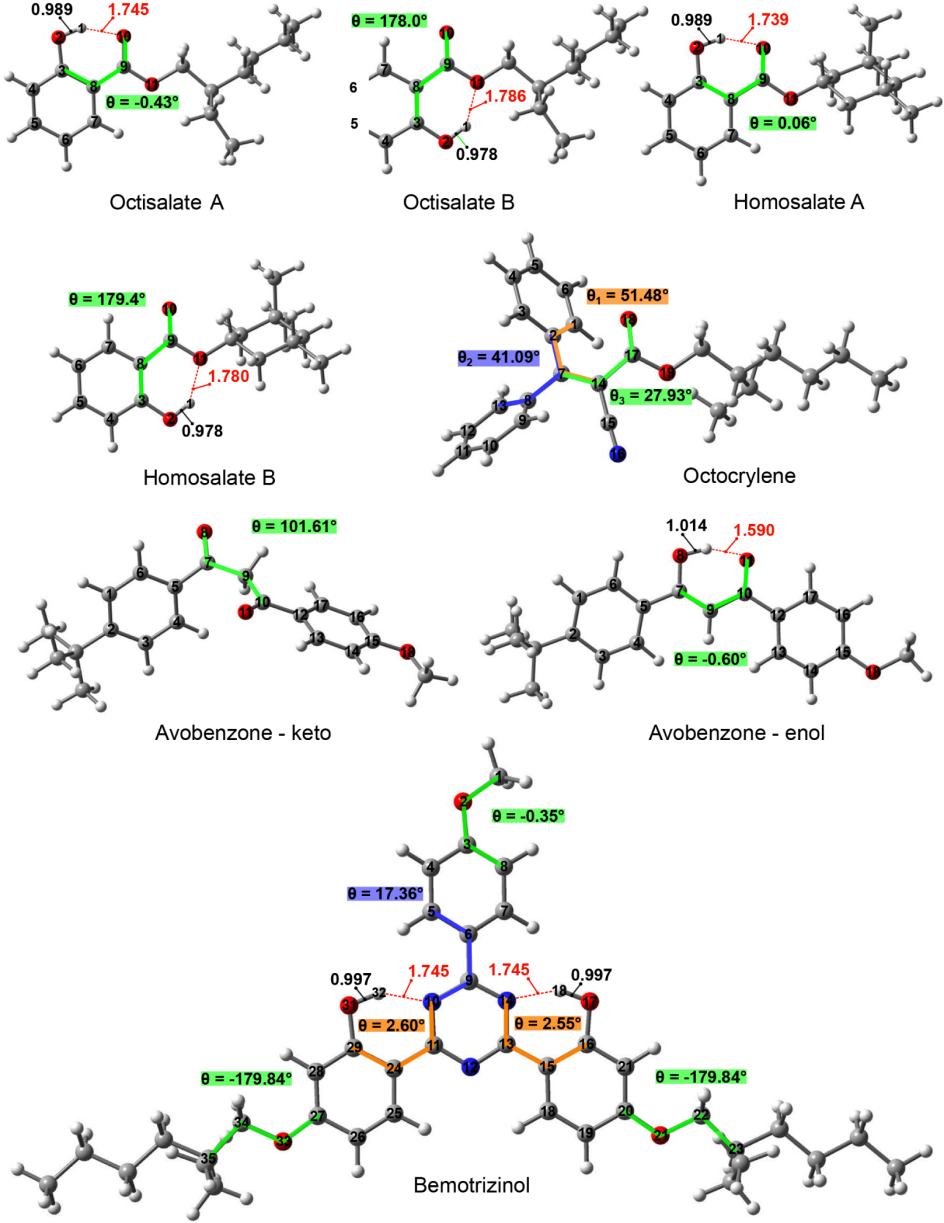
Relaxed singlet ground state (S_0_) geometries in the gas phase over the DFT/B3LYP/6-31+G­(d) approach. Bond lengths are given in Å, and dihedral angles are in degrees.

The difference in the total energy between A and B Octisalate molecules was −3.57 kcal mol^–1^ for the A conformer. Similarly, the difference in energy between A and B Homosalate conformers was −3.36 kcal mol^–1^, reinforcing the higher stability profile of the A conformer. The lengths of the O(2)–H(1) bonds are on the order of 0.978 and 0.989 Å in the conformational molecules B and A, respectively, evidencing the H(1)–O(6) bond in the pseudocycle of A. The Octocrylene (Figure 1) presented 51.48, 41.09, and 27.93° for dihedral angles among the benzyl groups and the saturated carbon chain. Avobenzone molecules have tautomerism through enol- and keto-conformers (Figure 1). The keto-conformer shows 101.61°, while the enol-conformer has −0.60° for the dihedral angle. The hydrogen bond in enol form is 1.590 Å, stretching the H–O bond to 1.014 Å. Furthermore, the difference in energy of 3.834 kcal mol^–1^ showed the enol conformation as more stable. There is a high molecular conjugation in the Bemotrizinol molecule around the central triazinic ring, described by 17.36, 2.60, and 2.55° with equal hydrogen bonds of 1.745 Å. The dihedral angles in peripheral groups are −0.35, −179.84, and −179.84°.

### Charge Analysis

In Table 1, all molecules exhibited high dipole moments (μ) in the solvated medium compared to the gas phase due to interactions between solute and solvent. These values reflect the polarity of the solvents. The μ values are larger for solvents with higher polarity and dielectric constants, while smaller values are observed in less polar solvents. Such results reflect the solvent’s strength to cause more charge separation. The Octisalate B and Homosalate B molecules present hydrogen bonds. However, the opposite charges on oxygen atoms determine the low μ. Bemotrizinol and Avobenzone-enol have large peripheral chemical groups with nonlinear charges localized on hydroxyl groups in the geometry center, creating polarizable places for solvent interactions. In another way, Octisalate A, Octocrylene, and Homosalate A demonstrate hydrogen bonds and charge separations in specific areas of the molecular geometries regarding diffuse chemical groups. Such a condition promoted high polarization widely intensified by the solvent effect. The most considerable μ value is for Avobenzone-keto, where the absence of a hydrogen bond between oxygen atoms results in an intense and near charge location, yielding higher polarization through molecular geometry. Consequently, the solvent effect caused a drastic increase in the μ.

**Table 1:** Dipole Moments (in Debye) for Molecules in the Singlet Ground State (S_0_) Using the DFT/B3LYP/6-31+G­(d) Approach for the Gas Phase and Water, Methanol, and Ethanol Solvents

Molecule	Gas phase	Methanol	Ethanol	Water
Octisalate B	1.763	2.112	2.105	2.126
Homosalate B	1.779	2.215	2.206	2.234
Bemotrizinol	2.357	3.360	3.339	3.401
Avobenzone-enol	2.949	4.404	4.375	4.460
Homosalate A	3.129	4.213	4.193	4.251
Octocrylene	3.199	4.752	4.722	4.810
Octisalate A	3.230	4.252	4.234	4.287
Avobenzone-keto	5.168	7.537	7.482	7.646

Figure 2 shows the electrostatic potential maps in which the regions in red and blue represent areas of high and low electron density, respectively. In A and B Octisalate, the regions with the highest electron density are above the oxygen atoms O(2) and O(10). The same behavior is observed for A and B Homosalate. Positive and neutral charges predominate in the saturated carbonic chain. While in the aromatic ring, there is a partial distribution of electrons representing the electronic resonance.

**Figure 2:**
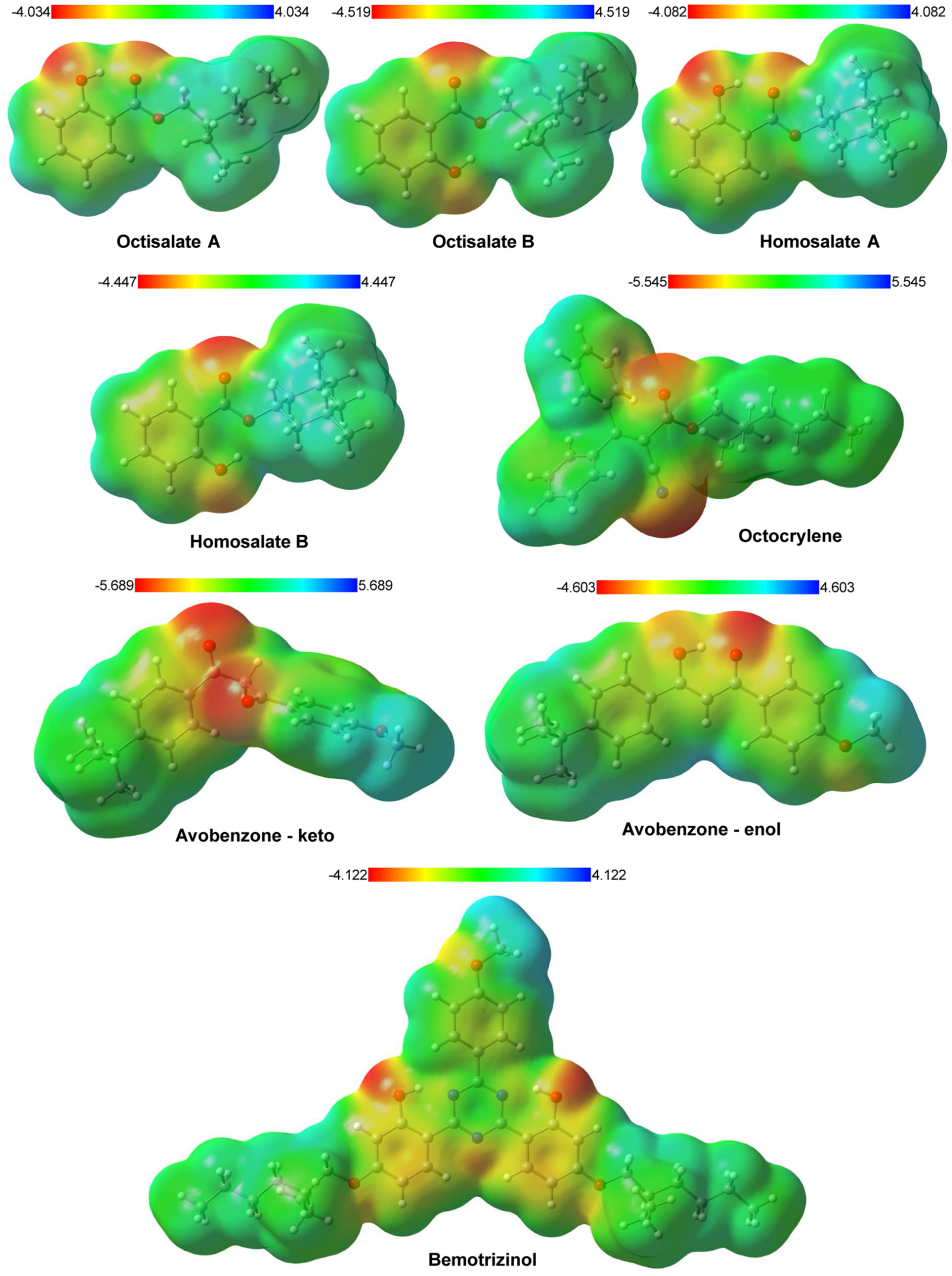
Electrostatic potential maps of UV filters: Homosalate A and B, Octisalate A and B, Avobenzone in keto and enol forms, Octocrylene, and Bemotrizinol. The values are in units of 10^–2^ au. Red regions indicate higher electron density, while blue regions correspond to lower density.

In Octisalate A and Homosalate A, there are two highly negative sites in different regions on oxygen atoms O(2) and O(10) due to the twisting of the aromatic ring. Furthermore, for two molecules B, such sites are close to each other, resulting in different interactions between the molecules and the solvents. In Octocrylene, the highest electronic density is localized on the oxygen atom, O(18) of the carbonyl, and the nitrogen atom, N(16) of the nitrile group. There is a neutral charge region on the benzyl rings featured by a diffuse electronic density through π resonance and acidic hydrogen. The ethylhexane group exhibits a neutral charge distribution characteristic of alkane groups. For the keto form, the O(8) and O(11) atoms exhibit greater negative density. The charge distribution in the methoxybenzyl group indicates the behavior of an electron-donating group. At the same time, the *tert*-benzyl group shows a profile of an electron-withdrawing group. Then, the misalignment between oxygen atoms in the keto form plays a crucial role in increasing the dipole moment of the molecule, resulting in a high charge transfer. The alignment of oxygen atoms through a hydrogen bond in the enol form of the Avobenzone molecule causes an electronic redistribution compared to the keto form. The hydrogen bond contributes to the formation of a pseudocycle, which stabilizes the molecule and creates electronic delocalization throughout the conjugated system. There is a region of higher electronic density among the O(8), O(11), and O(18) atoms, with a predominance over the O(11) atom. A small electronic density is distributed along the pseudocycle, causing high conjugation among the rings of the molecule.

In the methoxy group, the electron density is low, mainly concentrated on the O(18) atom. However, a charge transfer occurs in the methoxy group due to the formation of a pseudocycle. At the same time, the molecule exhibits a neutral charge on the benzyl group and a low positive region on the *tert* group. In Bemotrizinol, the highest electronic density regions are concentrated over the O(17) and the O(31) atoms. The methoxy group shows a positive region on the methyl group and a partial negative charge on the O(2) atom. The triazinic ring remained neutral overall. The N atoms connected by hydrogen bonds to hydroxyl groups are slightly positively charged. The N(12) atom presented a negative charge due to the strong interaction between the isolated electron pair and the hydrogen atoms of the aromatic rings. In aromatic rings, especially in the lower ones, electron density appears with greater intensity due to the resonance effect. Branched chains exhibit predominantly positive or neutral regions.

### Ultraviolet Absorption Spectra

Figure 3 shows the UVA and UVB optical absorption spectra calculated from the TD-DFT/6-31+G­(d) approach in a vacuum as well as in water, methanol, and ethanol, which are protic polar solvents. While some molecules displayed stronger absorption bands in the UVC region, the present analysis focuses solely on the peaks relevant to the UVA and UVB spectral ranges. The Octisalate and Homosalate optical absorption spectra are indistinguishable visually. The visual difference lies in the intensity of absorbance. The A conformers of salicylate derivatives showed absorption peaks near those observed in the B conformers. For Octisalate A (Figure 3a), the peaks were observed at 294.47 nm (*f* = 0.1114) in the gas phase, assigned to the HOMO → LUMO (69%) and HOMO – 1 → LUMO + 1 (16%) transitions (Figure S1). In solvents, peaks appeared at 293.71 nm (*f* = 0.1302) in water, 293.78 nm (*f* = 0.1302) in methanol, and 293.99 nm (*f* = 0.1322) in ethanol, all assigned to the HOMO → LUMO (69%) and HOMO – 1 → LUMO + 1 (15%) transitions.

**Figure 3:**
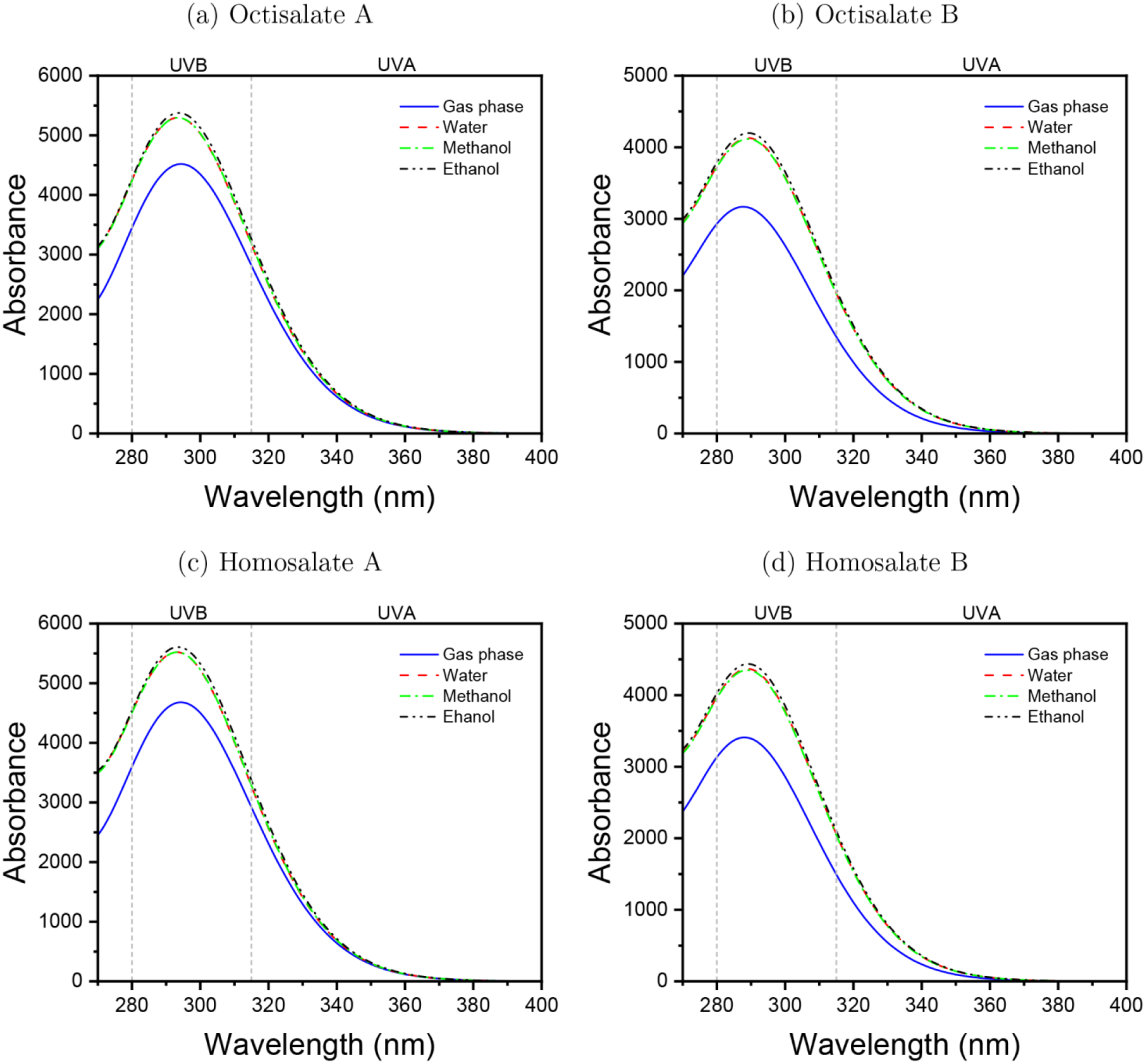
UVA and UVB optical absorption spectra calculated using the TD-DFT/B3LYP/6-31+G­(d) quantum approach. Octisalate A (a), Octisalate B (b), Homosalate A (c), Homosalate B (d). The gas phase in the blue line, water in the red dashed line, methanol in the green dash-dot line, and ethanol in the black dash-dot-dot indicate the protic polar solvents.

Furthermore, the Octisalate B (Figure 3b) showed absorption peaks at 288.06 nm (*f* = 0.0778) in the gas phase, due to the HOMO → LUMO (68%) and HOMO – 1 → LUMO + 1 (17%) transitions (Figure S2), and at 289.71 nm (*f* = 0.1011; water), 289.69 nm (*f* = 0.1008; methanol), and 289.83 nm (*f* = 0.1028; ethanol), all assigned to the HOMO → LUMO (68%) and HOMO – 1 → LUMO + 1 (16%) transitions. Nevertheless, the Homosalate A (Figure 3c) presented values of 294.49 nm (*f* = 0.1153; gas phase) (Figure S3), 293.56 nm (*f* = 0.1355; water), 293.63 nm (*f* = 0.1355; methanol), and 293.84 nm (*f* = 0.1376; ethanol). In all cases, the transitions involved were identical to those observed for Octisalate A, with equivalent contributions from the HOMO → LUMO and HOMO – 1 → LUMO + 1 excitations. Moreover, the Homosalate B (Figure 3d) exhibited peaks at 288.46 nm (*f* = 0.0837) for the gas phase (Figure S4), associated with HOMO → LUMO (68%) and HOMO – 1 → LUMO + 1 (17%) transitions, and 289.62 nm (*f* = 0.1068; water), 289.58 nm (*f* = 0.1064; methanol), and 289.73 nm (*f* = 0.1085; ethanol), all likewise assigned to the HOMO → LUMO (68%) and HOMO – 1 → LUMO + 1 (16%) transitions.

Experimental results indicate that Octocrylene (Figure 4a) exhibits an absorption peak near 303 nm,^57^ with variations according to the solvent nature.^32^ In the gas phase, Octocrylene showed a λ_max_ at 335.61 nm (*f* = 0.2609), associated with the transitions HOMO → LUMO (67%), HOMO – 1 → LUMO (13%), and HOMO – 2 → LUMO (13%) (Figure S5). In an aqueous medium, λ_max_ was observed at 345.02 nm (*f* = 0.3468), assigned to the HOMO → LUMO transition (69%). In methanol and ethanol, these peaks were observed at 344.84 nm (*f* = 0.3465) and 345.15 nm (*f* = 0.3512), respectively, both of which are also related to the HOMO → LUMO transition (69%). In the gas phase, the keto (Figure 4b) and enol (Figure 4c) forms of Avobenzone exhibit absorption peaks at 269.86 nm (*f* = 0.4667) and 342.76 nm (*f* = 0.9912), respectively. The 269.86 nm peak (*f* = 0.4667) is connected with the HOMO → LUMO (50%) and HOMO → LUMO + 1 (39%) (Figure S6). While the peak at 342.76 nm is associated with the HOMO → LUMO transition (70%) (Figure S7), whose frontier orbitals are distributed homogeneously throughout the molecule. In aqueous medium, the maximum absorptions were at 285 nm (*f* = 0.6481) from the HOMO → LUMO (70%) transition for the keto form, and at 360.86 nm (*f* = 1.1342), regarding the HOMO – 1 → LUMO (13%) and HOMO → LUMO + 1 (68%) transitions for the enol form. In methanol, the maximum absorption occurred at 284.64 nm (*f* = 0.6467), also related to the HOMO – 1 → LUMO (13%) and HOMO → LUMO + 1 (68%) transitions, and at 360.38 nm (*f* = 1.1318), similarly associated with the HOMO → LUMO (70%) transition. In ethanol, the maximum absorption peaks were observed at 284.79 nm (*f* = 0.6508) for the keto form, associated with the HOMO – 1 → LUMO (13%) and HOMO → LUMO + 1 (68%) transitions, and at 360.79 nm (*f* = 1.1378) for the enol form, mainly related to the HOMO → LUMO (70%) transition.

**Figure 4:**
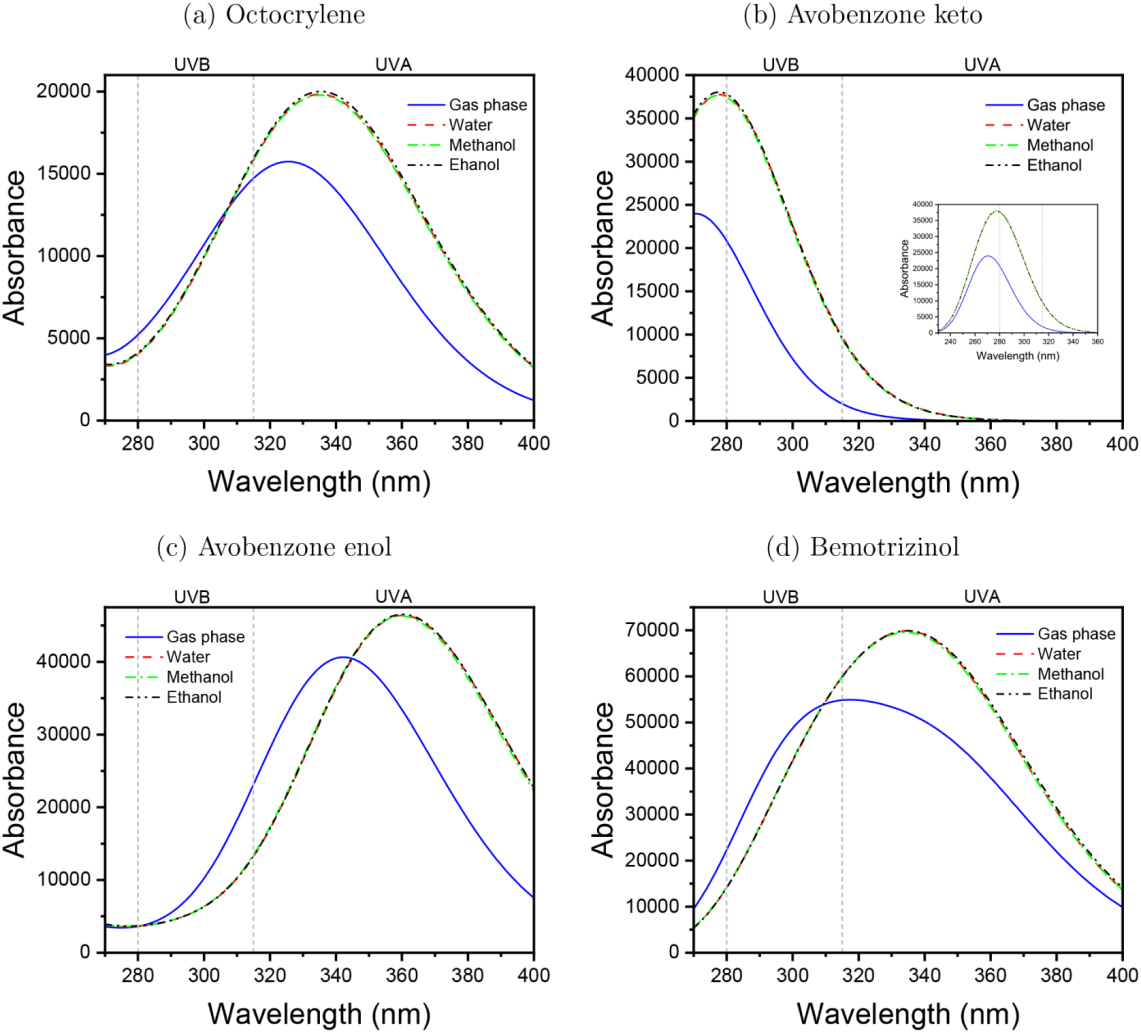
UVA and UVB optical absorption spectra calculated using the TD-DFT/B3LYP/6-31+G­(d) quantum approach. Octocrylene (a), Avobenzone-keto (b), Avobenzone-enol (c), Bemotrizinol (d). The vacuum in the blue line, water in the red dashed line, methanol in the green dash-dot line , and ethanol in the black dash-dot-dot indicate the protic polar solvents.

Among the UV filters, BEMT showed the highest simulated absorption intensities for both the gas and solvent phases. According to Herzog et al.,^58^ BEMT exhibits λ_max_ at 310 and 343 nm in ethanol. In the gas phase, BEMT exhibited the most intense absorption peak at 348.51 nm (*f* = 0.8151), associated with transitions from HOMO – 1 → LUMO (31%) and HOMO → LUMO + 1 (62%) (Figure S8). Two other notable peaks were identified at 301.11 nm (*f* = 0.5776) and 303.57 nm (*f* = 0.2242). In water, the λ_max_ shifted slightly to 348.99 nm (*f* = 1.0027), assigned to HOMO – 1 → LUMO (39%) and HOMO → LUMO + 1 (58%), accompanied by transitions at 311.28 nm (*f* = 0.5151) and 334.10 nm (*f* = 0.3825). In methanol, the main absorption was at 349.07 nm (*f* = 1.0005), with contributions from HOMO – 1 → LUMO (38%) and HOMO → LUMO + 1 (58%) transitions with additional peaks at 311.12 nm (*f* = 0.5224) and 334.08 nm (*f* = 0.3749). Similarly, in ethanol, the maximum peak occurred at 349.56 nm (*f* = 1.0085), assigned to HOMO – 1 → LUMO (38%) and HOMO → LUMO + 1 (58%), accompanied by peaks at 311.28 nm (*f* = 0.5271) and 334.33 nm (*f* = 0.3787). The electronic densities involved in the HOMO – 1 → LUMO and HOMO → LUMO + 1 transitions are mainly localized over the triazine core and aromatic rings without any significant contribution from the branched chains.

The investigated polar solvents (Table 2) promoted a red shift of the UVB absorption peak, in agreement with the experimental data reported by Holt et al.^59^ for Homosalate, and consistent with the minimal solvatochromic shifts commonly reported for similar molecules in polar environments.^27,28,59–61^ However, regarding the absolute value, the Homosalate and Octisalate UVB filters have close absorption profiles, with experimental absorption peaks around 307 nm,^59,62,63^ depending on the solvent used. Then, molecular factors absent in our models, such as the addition of the solvent molecules to explicitly model the solvent effect on the molecules, solute molecular geometry conformation, and solute–solute effects, can cause the deviation to red shift between experimental and theoretical results through the interaction with the electronic density of the solute. At the same time, the hydrogen bond on the A conformers caused a red shift compared to that of the B conformers. According to Beyere et al.,^28^ the hydrogen bond between solvents and solutes stabilizes the electronic structure, decreasing the energy of the electronic states and triggering the bathochromic effect.

**Table 2:** UVA and UVB Maximum Optical Absorption Wavelength Calculated Using TD-DFT/B3LYP/6-31+G­(d) Quantum Approach and Organized by Protic Polar Solvents

Molecule	Gas phase	Methanol	Ethanol	Water
Avobenzone-enol	342.76	360.38	360.79	360.86
Bemotrizinol	348.51	348.07	349.56	349.99
Octocrylene	335.61	345.84	344.15	345.02
Octisalate A	294.47	293.78	293.99	293.71
Homosalate A	294.49	293.63	293.84	293.56
Octisalate B	288.06	289.69	289.83	289.71
Homosalate B	288.46	289.58	289.73	289.62
Avobenzone-keto	269.86	285.64	284.79	284.00

Avobenzone exhibits keto–enol tautomerism, with the UV absorption directly connected to the tautomer.^60,64,65^ While the keto tautomer absorbs mainly in the UVB region, the enol tautomer is responsible for absorption in the UVA region.^66^ In the UVA region, the maximum absorption peak appears between 350 and 365 nm, depending on the solvent used. The reported peak values include 354.9 nm,^60^ 356 nm,^15^ and 358 nm^27,66^ in ethanol. Very close peaks of 355 nm^60^ and 356 nm^15^ were observed in acetonitrile. For nonpolar solvents, such as hexane and cyclohexane, the peaks shift to 350 nm^60^ and 351 nm,^27^ respectively. In DMSO, a highly polar solvent, peaks are reported at 363 nm^27^ and 364 nm.^15^

The bathochromic effect, characterized by a red shift, indicates a correlation between UV absorbance and the solvent dielectric moments, which are connected to the molecular dipole moments. Figure 5 presents such a correlation in a vacuum and protic polar solvents. The solvent effect increased the dipole moments in accordance with the molecular charge separation. For the molecular group composed of Octisalate A, Homosalate A, Octisalate B, Homosalate B, and Bemotrizinol, there is an increase in the dipole moment, accompanied by a slight red shift of 1 nm. In another way, the Avobenzone-keto, Octocrylene, and Avobenzone-enol had an increase in the dipole moment followed by a significant red shift from 10 to 18 nm. The red shift was more critical for Avobenzone-keto, changing the light absorbance from UVC to UVB. The strong charge separation promoted by protic polar solvents indirectly influenced the electronic transition because of the force competition on the electronic density. The external electrostatic force field induced an ″extra″ molecular interaction component to displace the electronic density in the excited state during the electronic transition. In this field, the electronic transition is affected, resulting in a variation in UV absorbance related to the dipole moment.

**Figure 5:**
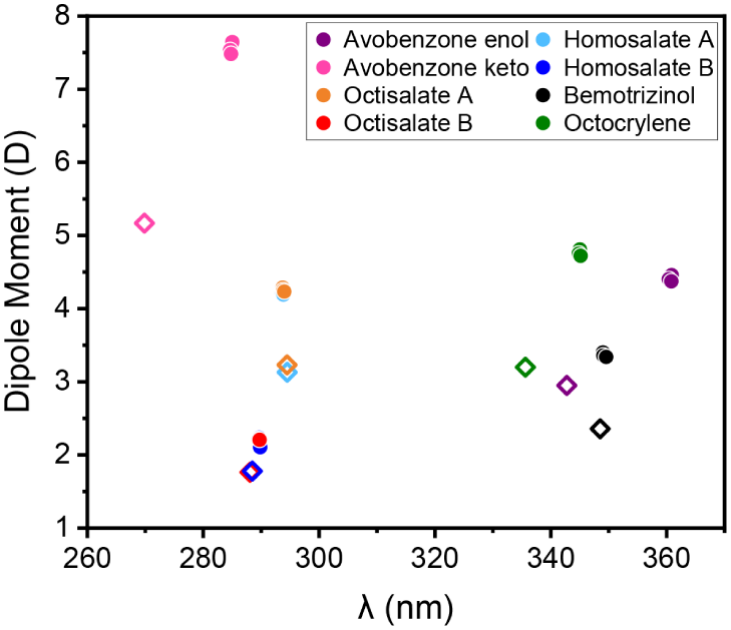
UV organic filters organized according to absorption peaks (in nm) and dipole moments (in debye). The empty squares represent the molecules in the gas phase, and the filled circles represent the solvated molecules.

### Solvent Effect

The prediction of the solvated energies applying the PCM from DFT calculations in molecules is a representation that is largely disseminated. The Gibbs energy used for the solvent effect is determined through the energy difference between vacuum and PCM simulations, Δ*G*_solv_ = *G*_gas phase_ – *G*_PCM_. At this point, Δ*G*_solv_ represents the insertion of the molecule from the gas phase (vacuum) into the solvent medium. Subsequently, there is a relaxation between the molecule and the electric field, allowing the solvent effect to deform the ground-state gas-phase geometry. Then, the calculated Δ*G*_solv_ values are referenced to relaxed molecules under the solvent effect. The electronic total energies used to calculate the solvent Gibbs energies are given in the Supporting Information.

Table 3 presents the Δ*G*_solv_ energies for the solved molecules under water, methanol, and ethanol solvents through PCM simulation. The solvent effects indicate favorable Gibbs energies in all of the solvents. Molecules without hydrogen bonds, such as Avobenzone-keto, Octocrylene, and Bemotrizinol, had the highest energies. Molecules with hydrogen bonds through the saturated oxygen, such as Avobenzone-enol, Octisalate B, and Oxazolate B, exhibit intermediate energies. Furthermore, the hydrogen bonds formed through unsaturated oxygen, such as those in Octisalate A and Omosalate A, yielded the lowest energies. These molecular groups separate the solvent effect through molecular geometries, suggesting the predominance of an inductive effect.

**Table 3:** Solvent Gibbs Energies (Δ*G*_solv_ in kcal·mol^–1^) of the Avobenzone, Keto and Enol, Homosalate A and B, Octisalate A and B, and Octocrylene and Bemotrizinol Simulated under Water, Methanol, and Ethanol Solvents

Molecules	Water	Methanol	Ethanol
Avobenzone-keto	–10.65	–10.33	–10.16
Octocrylene	–10.06	–9.76	–9.62
Bemotrizinol	–9.39	–9.07	–8.91
Avobenzone-enol	–7.28	–7.04	–6.94
Octisalate B	–6.23	–6.21	–6.25
Homosalate B	–5.83	–5.63	–5.53
Octisalate A	–4.92	–4.77	–4.69
Homosalate A	–4.90	–4.74	–4.65

### Molecular Dynamics

The Gibbs energies calculated from the DFT simulations indicate the favorable or unfavorable solvent effects on the Avobenzone, Octocrylene, Octisalate, Homosalate, and Bemotrizinol molecules. However, the DFT solvent simulations were performed on a single molecule under an electric field. In another way, the quantity of molecules or solutes is an essential factor regarding the solvent effect. Then, the analysis of discrete DFT solvent and MD aggregation states significantly contributes to understanding molecular behavior under different solvent conditions.

Figure 6 shows the aggregation states in water of the keto- and enol-Avobenzone, Homosalate B, Octisalate A, Octocrylene, and Bemotrizinol molecules simulated by molecular dynamics. Avobenzone-keto (Figure 6a) molecules exhibited a partial aggregation state, indicating dipole–dipole interactions among the molecules. In comparison, the Avobenzone-enol (Figure 6b) shows a low aggregation state, characterized by more interaction with the solvent than with other Avobenzone-enols. The dipole moment is strictly localized. Furthermore, Homosalate B (Figure 6c) and Octisalate A (Figure 6d) indicated aggregation states organized in pairs, suggesting more specific molecular interactions such as induced dipoles. For Octocrylene (Figure 6e) and Bemotrizinol (Figure 6f), the agglomeration among the molecules is more intense, creating structures similar to micelles due to the high electronic densities distributed on the bulky chemical groups at the periphery of the molecule.

**Figure 6:**
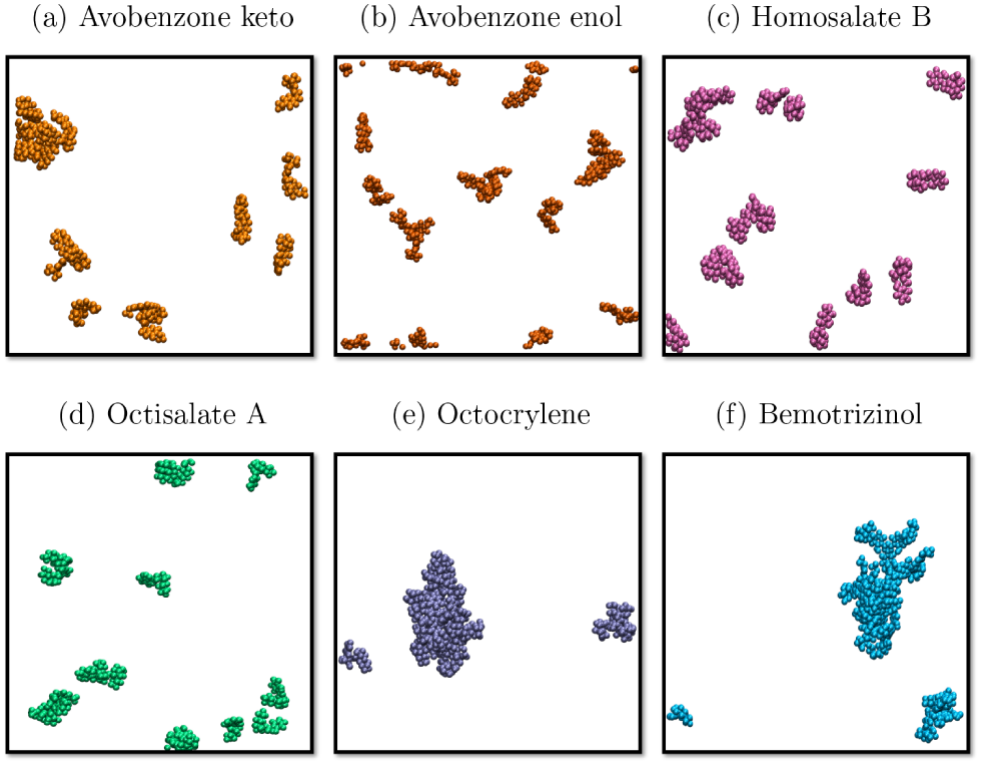
Aggregation states simulated using molecular dynamics in water solvent: (a) Avobenzone-keto, (b) Avobenzone-enol, (c) Homosalate B, (d) Octisalate A, (e) Octocrylene, and (f) Bemotrizinol. Water molecules omitted.

To corroborate these initial impressions, volumetric profiles (Figure 7) were performed according to the dimensions of the box and over a simulation time interval of 5.0 ns. The Avobenzone-keto (Figure 7a), Octocrylene (Figure 7b), and Bemotrizinol (Figure 7c) present volumetric structures with the agglomeration of molecules. For the other molecules, Avobenzone-enol (Figure 7d), Octisalate A (Figure 7e), and Homosalate B (Figure 7f), they show low volumetric density, indicating less aggregation.

**Figure 7:**
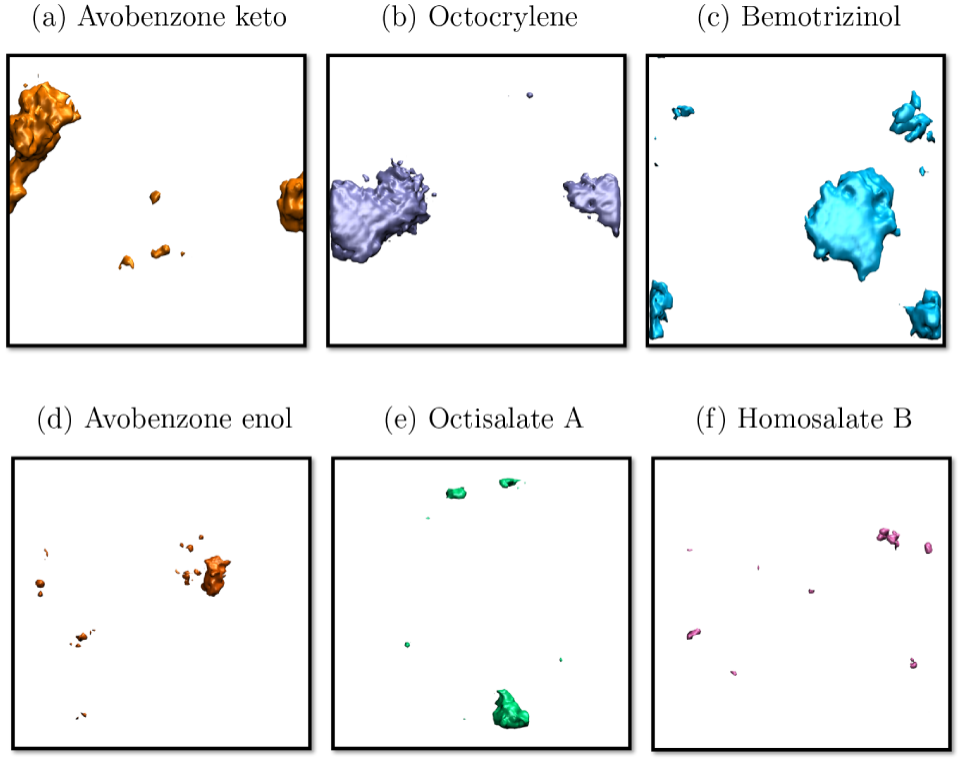
Volumetric density profiles simulated using molecular dynamics in water solvent. (a) Avobenzone-keto, (b) Octocrylene, (c) Bemotrizinol, (d) Avobenzone-enol, (e) Octisalate A, and (f) Homosalate B. Water molecules omitted.

Another analysis to complement the discussion on aggregated molecules was the density profile along the *z*-axis (Figure 8). A constant straight line in the density profile represents an ideal system, indicating a homogeneous solution fully diluted with dispersed molecules. In contrast, lines with high density variation, similar to broad peaks, represent a heterogeneous solution or a poorly diluted one, more associated with aggregated molecules. Figure 8a shows the density profiles for Avobenzone-keto, Octocrylene, and Bemotrizinol molecules. Avobenzone-keto presented a low-heterogeneous solution with an intermediate dilution. However, the Bemotrizinol and Octocrylene molecules exhibited more insoluble behavior, as suggested by the highly heterogeneous solution and low dilution, which favors aggregation. Furthermore, Figure 8b presents the density profiles for Avobenzone-enol, Octisalate A, and Homosalate B molecules. These results show a low variation in the density profiles along the *z*-axis, indicating a constant value. Consequently, the simulations represent a homogeneous solution with a high degree of molecular dilution. The Homosalate B molecules form the curve closest to the ideal solubility. The density profiles estimated the aggregation and solubility degrees, indicating the following sequence from the most (heterogeneous) to the least (homogeneous) aggregated states: Octocrylene > Bemotrizinol > Avobenzone-keto > Octisalate A > Avobenzone-enol > Homosalate B. Other radial distribution functions (RDFs) are provided in the Supporting Information (Figures S11, S12, S13), corroborating the density profiles.

**Figure 8:**
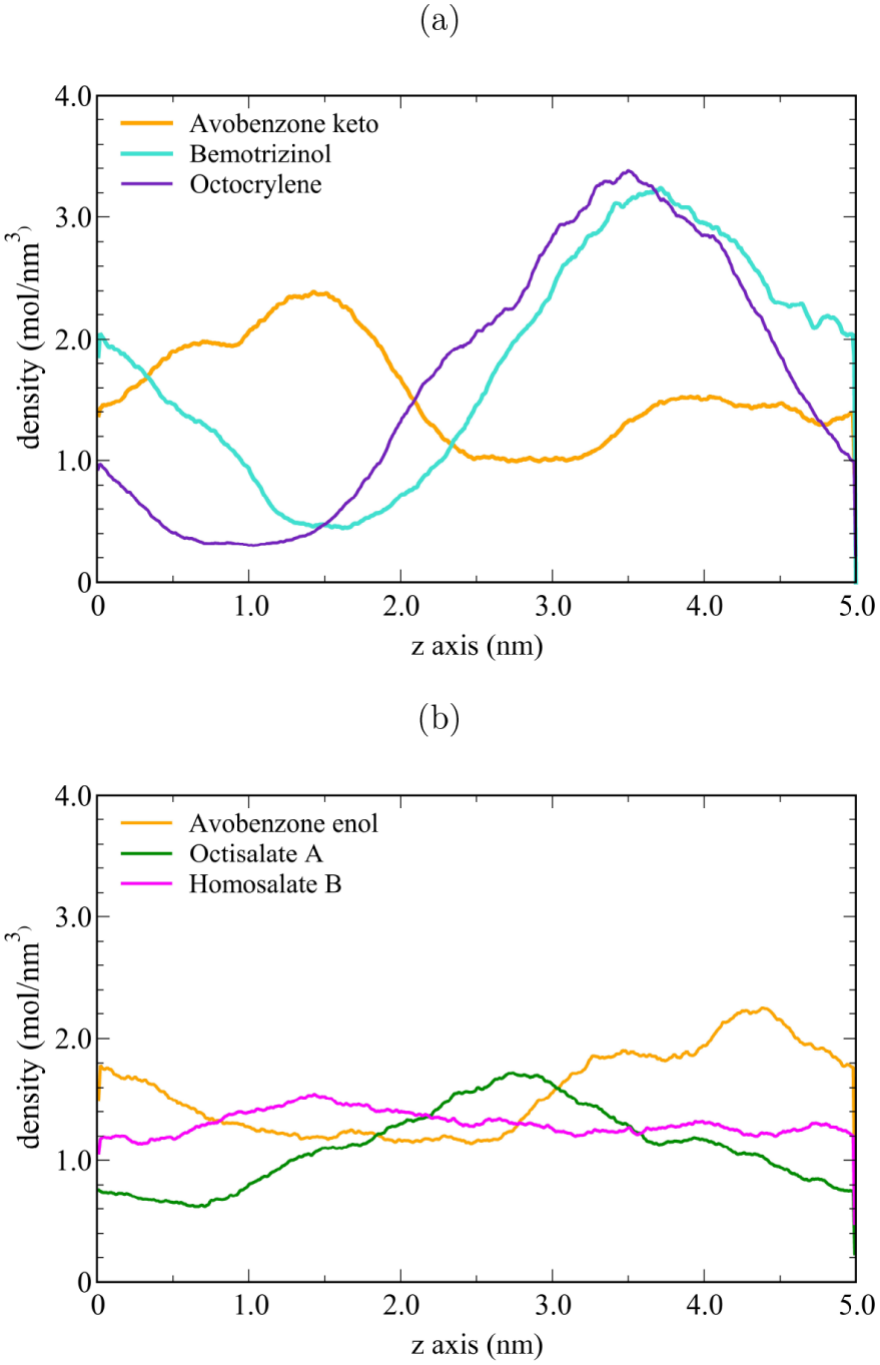
Density profiles performed along the *z*-axis using molecular dynamics in a water solution. Avobenzone-keto, Bemotrizinol, and Octocrylene (a); Avobenzone enol, Bemotrizinol, and Octocrylene (b).

## Conclusion

The solvent effect substantially changed the dipole moment and UV absorbance of the Avobenzone, Octocrylene, Octisalate, Homosalate, and Bemotrizinol molecules in a vacuum. The dielectric constants of water, methanol, and ethanol are very different; however, the changes in the dipole moment and UV absorbance were small. Molecular dynamics simulations simulated different aggregation states for the Avobenzone, Octocrylene, Octisalate, Homosalate, and Bemotrizinol molecules in the presence of water. Then, the combination of quantum and molecular dynamics models is a promising tool for studying optically active molecules, such as UV organic sunscreens, spanning discrete to continuum scales. The union between both simulation techniques can promote new insights for laboratory or industrial research.

## Future Scope

UV organic sunscreens are active, optically active molecules used for health protection and solar light absorption for energy conversion. The proposed research aims to align quantum and molecular dynamics approaches to evaluate synthetic or natural molecules and improve efficiency on both scales. The molecular profile will permit clarification of the molecular behavior in discrete and continuum scales. Sometimes, the solvent effect at the quantum level indicates favorable dissolution of a molecule; however, the quantity of molecules is a determinant for this effect. Then, molecular dynamics simulations explore such a possibility. In the next paper, results for methanol and ethanol solvents, not reported here, and molecules from natural sources are presented.

## Supplementary Material



## Notes

Energy diagrams of the Frontier Molecular Orbitals (FMOs), all relaxed molecular geometries in the gas phase, vacuum, 0 K, and singlet ground state using DFT approach (PDF)
