# Supplementary material for: In Silico Perspective on Avobenzone, Octisalate, Octocrylene, Homosalate, and Bemotrizinol as Organic UV Filters Using DFT, TD-DFT, and Molecular Dynamics
Source: ACS Omega. 2026 Feb 2;11(6):9369–80. doi: 10.1021/acsomega.5c09234 (PMC12917627; doi:10.1021/acsomega.5c09234)
Supplement: Supplementary file 1 [file ao5c09234_si_001.pdf]

In Silico Perspective on Avobenzone, Octisalate, Octocrylene, Homosalate, and Bemotrizinol Organic UV Filters Applying DFT, TD-DFT, and Molecular Dynamics

Maria E. Rigoni,† Sergio R. de Lazaro,*,† and Lucas S. de Lara‡

†*Department of Chemistry, State University of Ponta Grossa, Av. Carlos Cavalcanti 4748, Uvaranas, Ponta Grossa, Brazil*

‡*Department of Physics, State University of Ponta Grossa, Av. Carlos Cavalcanti 4748, Uvaranas, Ponta Grossa, Brazil*

E-mail: srlazaro@uepg.br

Table S1: Total Energies (in a.u.) for the Avobenzone-keto, Avobenzone-enol, Homosalate A and B, Octisalate A and B, Octocrylene, and Bemotrizinol, simulated under vacuum, water, methanol, and ethanol solvents. Energy conversion 1 a.u. = 627,509 kcal mol^{-1}.

Molecules	Vacuum	Water	Methanol	Ethanol
Avobenzone-keto	-1001.0870	-1001.1043	-1001.1037	-1001.1034
Avobenzone-enol	-1001.0931	-1001.1045	-1001.1041	-1001.1039
Homosalate A	-848.7014	-848.7082	-848.7080	-848.7078
Homosalate B	-848.6961	-848.7043	-848.7041	-848.7039
Octisalate A	-810.5843	-810.5912	-810.5910	-810.5909
Octisalate B	-810.5788	-810.5872	-810.5870	-810.5868
Octocrylene	-1136.0468	-1136.0624	-1136.0618	-1136.0616
Bemotrizinol	-2017.9922	-2018.0051	-2018.0047	-2018.0045

Frontier molecular orbitals

The following Figures show the Frontier Molecular Orbitals (FMO) analyzed in the Section Ultraviolet Absorption Spectra to discuss the absorption ways in the gas phase.

Figure S1: Representation of the HOMO-1, HOMO, LUMO, and LUMO+1 FMOs for the Octisalate A.

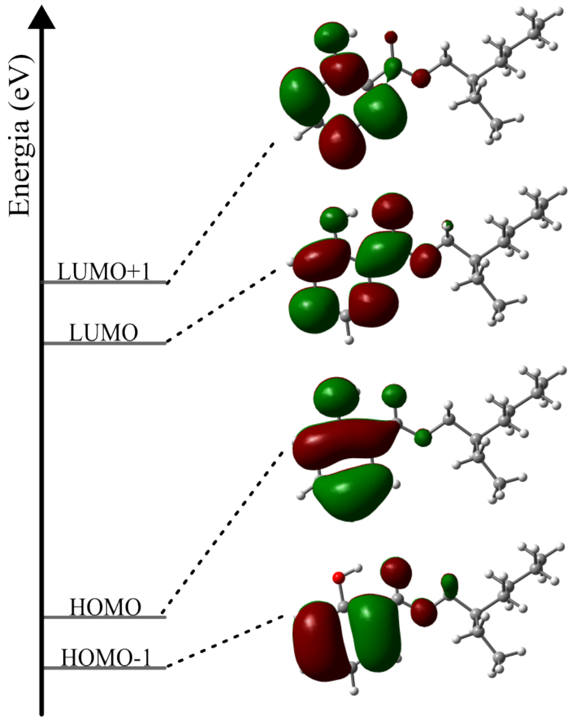

Figure S2: Representation of the HOMO-1, HOMO, LUMO, and LUMO+1 FMOs for the Octisalate B.

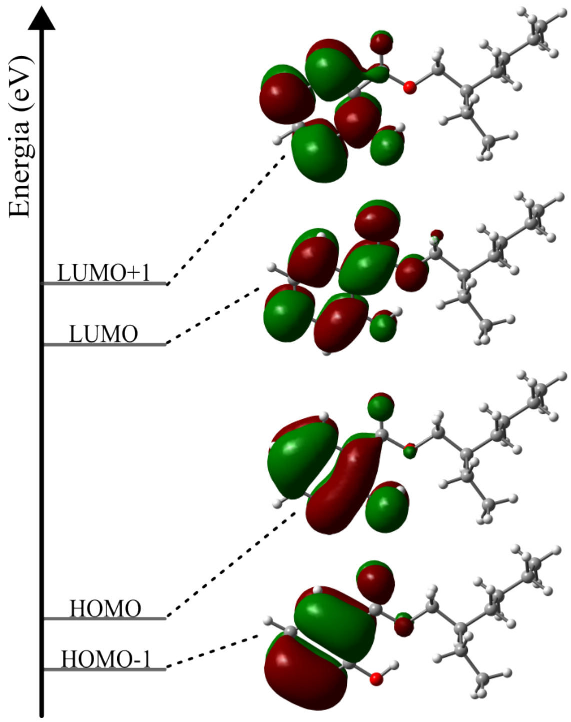

Figure S3: Representation of the HOMO-1, HOMO, LUMO, and LUMO+1 FMOs for the Homosalate A.

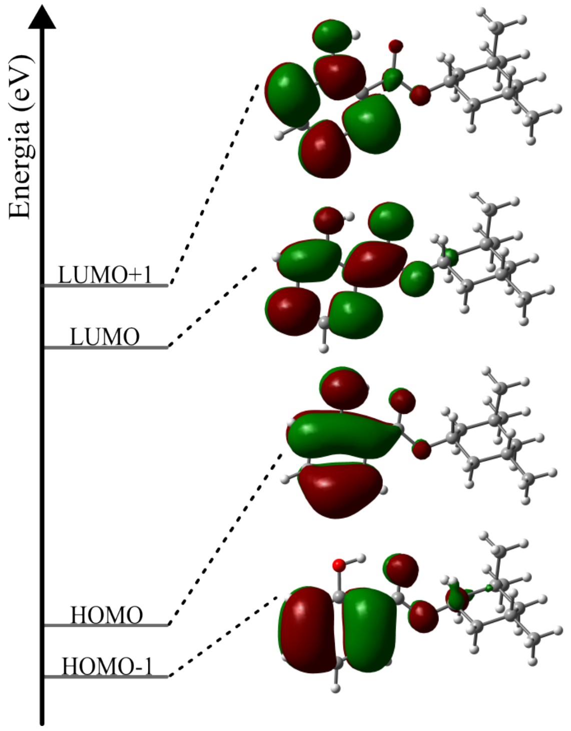

Figure S4: Representation of the HOMO-1, HOMO, LUMO, and LUMO+1 FMOs for the Homosalate B.

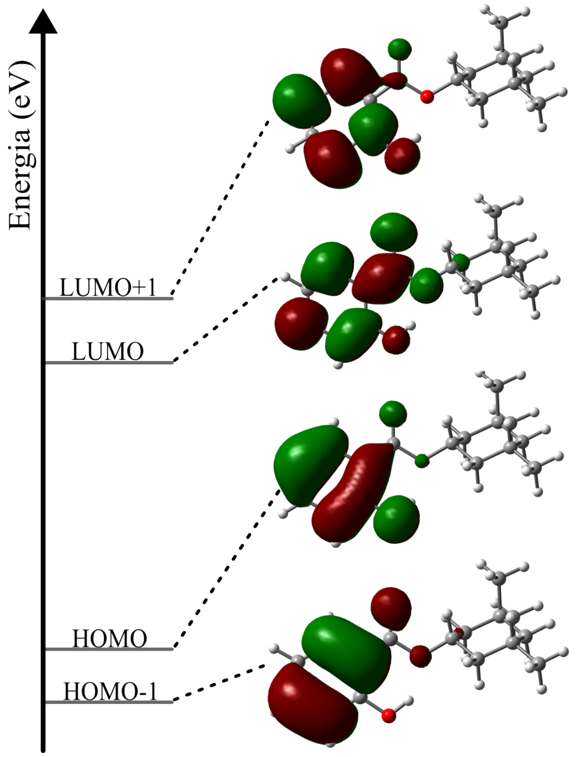

Figure S5: Representation of the HOMO-2, HOMO-1, HOMO, and LUMO FMOs for the Octocrylene.

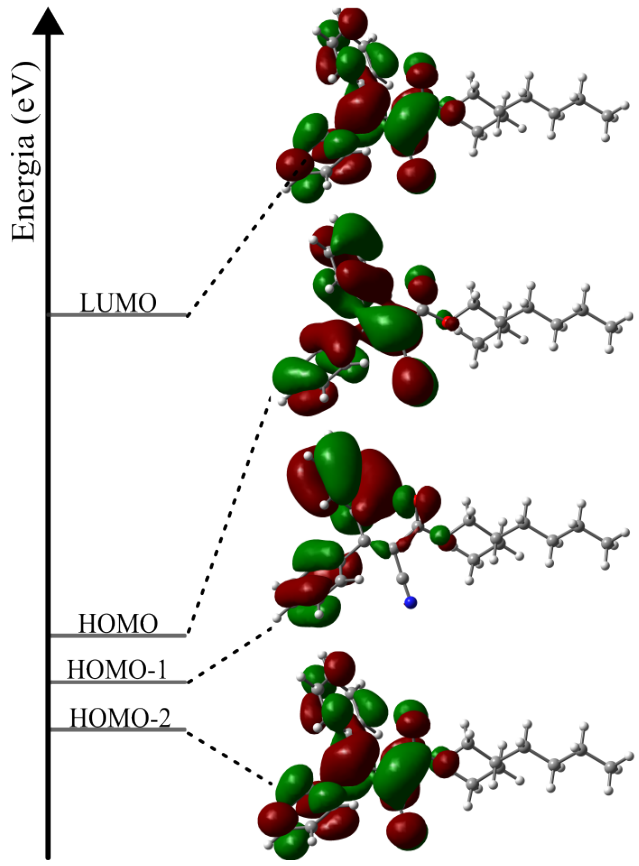

Figure S6: Representation of the HOMO-1, HOMO, LUMO, and LUMO+1 FMOs for the Avobenzone keto.

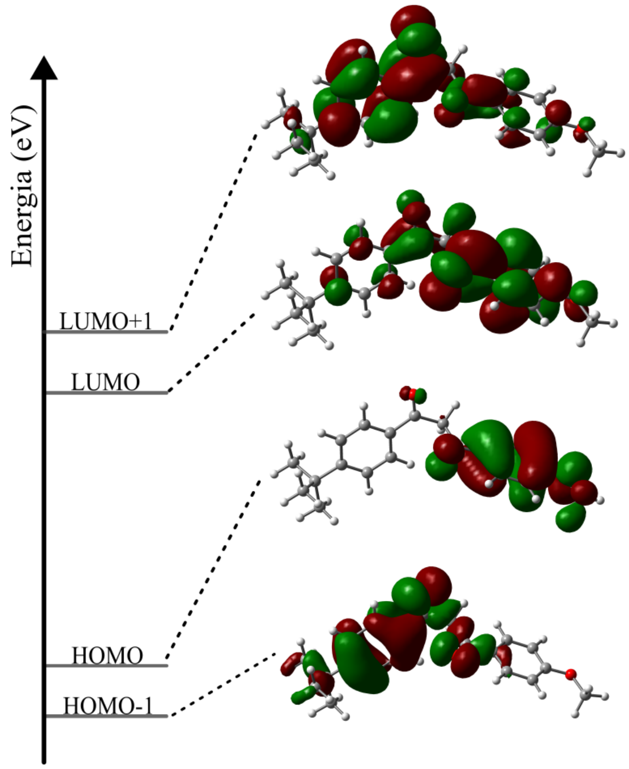

Figure S7: Representation of the HOMO and LUMO FMOs for the Avobenzone enol.

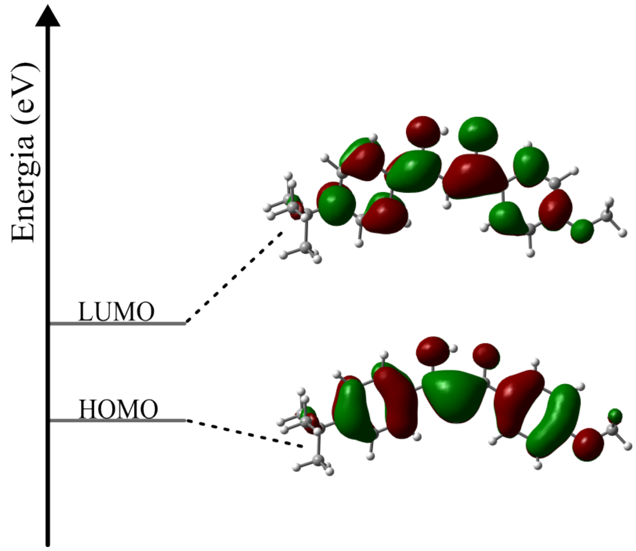

Figure S8: Representation of the HOMO-1, HOMO, LUMO, and LUMO+1 FMOs for the Bemotrizinol.

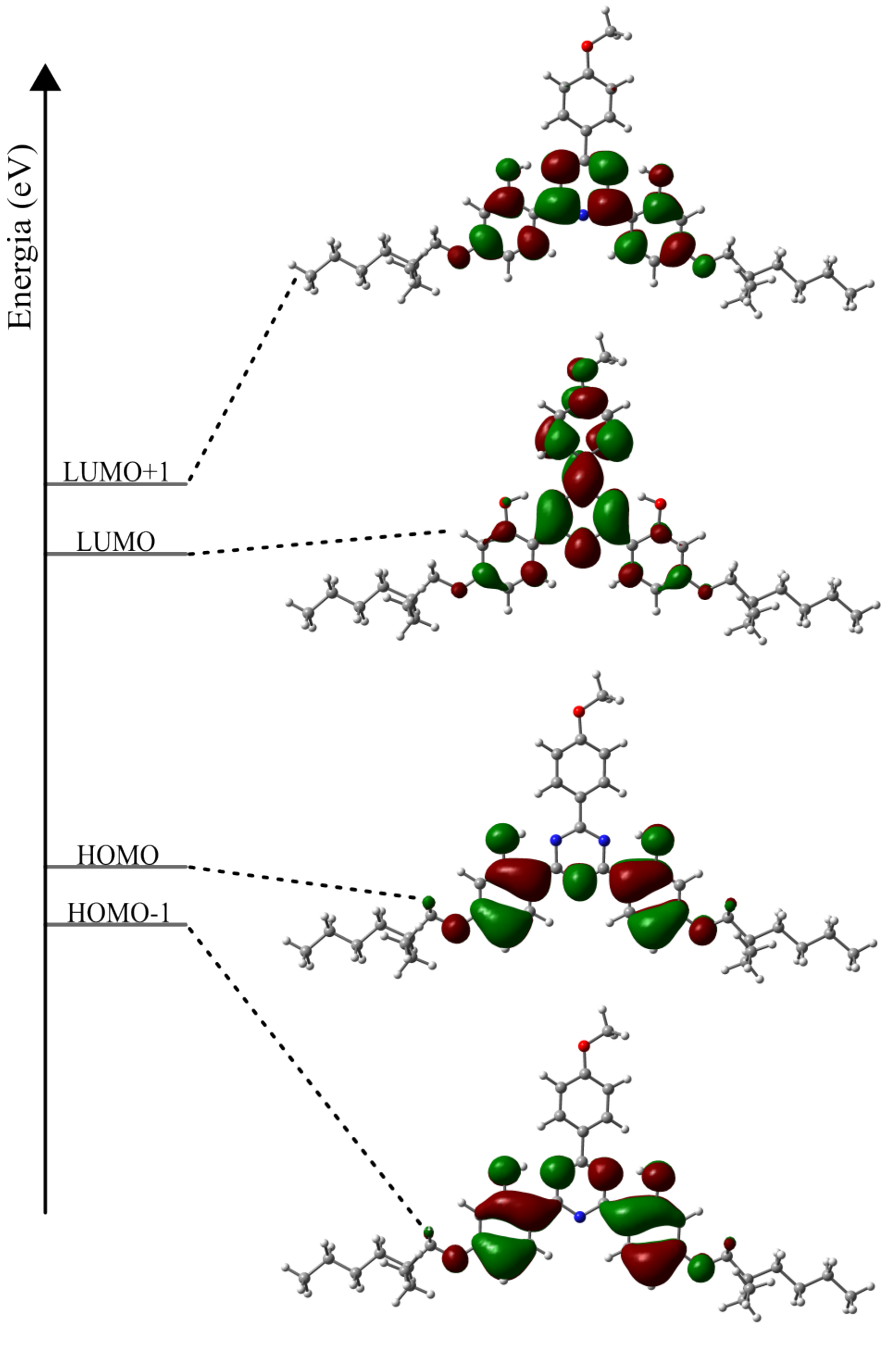

Quantum level

Relaxed molecular geometries calculated from DFT/B3LYP/6-31+(d) in singlet ground state, vacuum, zero Kelvin, and gas phase are below:

Avobenzone - keto

C	7.52638500	-1.76741200	0.33436500
H	7.69776200	-1.17656700	1.24272900
H	7.02349600	-2.70759300	0.59303700
H	8.48192000	-1.98478900	-0.14494100
O	6.77540100	-1.02915400	-0.62588000
C	5.52179600	-0.61375700	-0.30382100
C	4.84483200	0.11125000	-1.29931500
C	4.89245600	-0.86299400	0.92705600
C	3.55931200	0.57898100	-1.06531100
H	5.34879400	0.29546600	-2.24317400
C	3.60160100	-0.38842400	1.14546100
H	5.39575100	-1.41791400	1.71079300
C	2.91099000	0.33703600	0.16219100
H	3.06173100	1.13979800	-1.85066800
H	3.10552300	-0.57441600	2.09305900
C	1.53143700	0.81233300	0.46884400
C	0.79478200	1.63339000	-0.59767600
H	0.69668600	1.03492900	-1.51278900
H	1.39716900	2.51327400	-0.84964300
C	-0.56343800	2.15378700	-0.12519200
C	-1.75036000	1.25010300	-0.12648800
C	-1.69359500	-0.09319700	-0.51834100

C	-2.99180300	1.76805300	0.28208800
C	-2.83842300	-0.89377400	-0.50473700
H	-0.75378900	-0.54053800	-0.82774800
C	-4.12612700	0.96688600	0.29348200
H	-3.04364700	2.80724900	0.59176400
C	-4.08178200	-0.38656900	-0.09923900
H	-2.74385100	-1.92898300	-0.81330400
H	-5.06597200	1.40513800	0.61761600
O	0.99713700	0.56456700	1.54187800
O	-0.65679600	3.32413900	0.22127600
C	-5.36183900	-1.23927600	-0.06325100
C	-6.43184200	-0.60305000	-0.98449000
H	-7.35066500	-1.20252600	-0.96621800
H	-6.69127800	0.41342900	-0.67007800
H	-6.07866300	-0.55320900	-2.02138300
C	-5.90070200	-1.29165900	1.38785400
H	-6.81681400	-1.89430500	1.42950600
H	-5.16480900	-1.74306500	2.06366800
H	-6.13983900	-0.29434100	1.77205600
C	-5.12097500	-2.68609700	-0.53730400
H	-4.76469900	-2.72410100	-1.57364800
H	-4.39532500	-3.20985200	0.09612600
H	-6.06164900	-3.24715900	-0.49200300

Avobenzone - enol

C	8.10407500	-1.11168200	0.01594300
H	8.22416800	-0.54210300	-0.91425800

H	8.24484500	-0.44660200	0.87729000
H	8.84308000	-1.91362700	0.05010500
O	6.83532500	-1.75648800	0.06510400
C	5.70883600	-0.99245200	0.04027800
C	4.49344700	-1.69492100	0.09569900
C	5.69725400	0.40871400	-0.03423100
C	3.28914900	-1.00599600	0.07572000
H	4.52040300	-2.77878500	0.15492900
C	4.47875900	1.08527600	-0.05190500
H	6.62079800	0.97514300	-0.07814100
C	3.25483100	0.40104800	0.00102900
H	2.37057000	-1.58140300	0.12310200
H	4.45969100	2.16874700	-0.10789700
C	1.99783500	1.20120100	-0.02426400
C	0.70727300	0.55682200	-0.01659400
H	0.63970800	-0.51957900	0.02576200
C	-0.45959100	1.29557400	-0.04378200
C	-1.81988900	0.71914400	-0.03761300
C	-2.06171800	-0.64748400	-0.24287800
C	-2.92964600	1.55608000	0.17694200
C	-3.35921300	-1.15874300	-0.22590500
H	-1.23889700	-1.32848100	-0.43744600
C	-4.22005600	1.03636400	0.19538400
H	-2.76875600	2.61732400	0.33209300
C	-4.47329800	-0.33346100	-0.00343800
H	-3.49098000	-2.22158800	-0.39601400
H	-5.04705700	1.71941500	0.36936200

O	2.08592100	2.46088500	-0.05399600
C	-5.91748900	-0.86343500	0.02050900
C	-6.73456300	-0.17560800	-1.10098900
H	-7.76912100	-0.54138000	-1.09494400
H	-6.76433100	0.91215200	-0.97634900
H	-6.30421200	-0.38810600	-2.08679900
C	-6.56131100	-0.54389900	1.39227900
H	-7.59351700	-0.91489900	1.42070400
H	-6.00545500	-1.02092700	2.20815100
H	-6.58922800	0.53272400	1.59112000
C	-5.98961400	-2.38739600	-0.19869400
H	-5.58487500	-2.68136900	-1.17441300
H	-5.44862700	-2.93950100	0.57905600
H	-7.03582300	-2.71296100	-0.16576400
O	-0.42939200	2.62730100	-0.06602200
H	0.55235800	2.88133500	-0.06127700

Homosalate A

C	-5.04367200	-0.30004800	-0.00073100
C	-3.74917600	-0.70450900	-0.36760600
C	-2.63806800	0.10803600	-0.01898500
C	-2.86119600	1.30634700	0.68977600
C	-4.14320000	1.69806100	1.04816700
C	-5.23432800	0.88490900	0.69755300
H	-5.87738300	-0.93780200	-0.27820900
H	-2.00574800	1.91988500	0.95075800
H	-4.30023800	2.62351600	1.59421500

H	-6.24252500	1.18341500	0.97433000
O	-3.62710600	-1.86410600	-1.04363100
H	-2.66476500	-2.00440700	-1.22446700
C	-1.28350900	-0.32599500	-0.40511700
O	-1.05702600	-1.37140300	-1.02489900
O	-0.30975500	0.51499500	-0.02773300
C	1.07407500	0.16423700	-0.34811000
C	1.83272900	1.47782300	-0.50694000
C	1.65060500	-0.69838400	0.77599200
H	1.05496000	-0.38927700	-1.29005700
C	3.33451800	1.22870000	-0.74546000
H	1.70221300	2.07681100	0.40669300
H	1.40262800	2.05640200	-1.33449200
C	3.16272700	-0.99247600	0.59748100
H	1.49872900	-0.16065300	1.72282500
H	1.09134300	-1.63935100	0.84268500
C	3.91310500	0.34536200	0.37653600
H	3.44258400	0.69414800	-1.70107800
H	4.97322200	0.13693900	0.17284600
H	3.88697800	0.92131400	1.31519500
C	3.69024500	-1.66112800	1.88115900
H	3.17292100	-2.60951400	2.07525600
H	4.76317700	-1.87728000	1.79818100
H	3.54498200	-1.01415500	2.75558600
C	3.39783400	-1.95897800	-0.58396800
H	4.46634100	-2.18831900	-0.68515100
H	2.86678200	-2.90503900	-0.42181100

H	3.05655300	-1.55393500	-1.54239700
C	4.10101700	2.55275100	-0.86780600
H	5.16512200	2.37705600	-1.06810900
H	3.70353500	3.16931900	-1.68362700
H	4.02746500	3.13679400	0.05927300

Homosalate B

C	-4.45590100	1.08246100	0.81103600
C	-3.09859100	0.86451400	0.52304100
C	-2.71851900	-0.27430400	-0.23119400
C	-3.72414300	-1.15963000	-0.67191800
C	-5.06301000	-0.93839200	-0.38487200
C	-5.42411000	0.19324000	0.36336000
H	-4.71832900	1.96241600	1.39045400
H	-3.41269800	-2.02574500	-1.24712400
H	-5.82033800	-1.63349100	-0.73497400
H	-6.46918200	0.38058000	0.59745100
O	-2.22981000	1.78784700	1.00192600
H	-1.32452100	1.52948000	0.73566800
C	-1.32354900	-0.62357300	-0.59865800
O	-1.00017700	-1.60689000	-1.23683200
O	-0.40442600	0.28688800	-0.14584600
C	1.01359000	0.03969500	-0.42353500
C	1.69336200	1.40143300	-0.52123300
C	1.60512800	-0.82115600	0.69319200
H	1.05490600	-0.48374700	-1.38130600
C	3.21335100	1.24617300	-0.72362900

H	1.50855000	1.96504300	0.40618500
H	1.25269400	1.97921700	-1.34377700
C	3.13706900	-1.01945800	0.55515600
H	1.39216200	-0.32417600	1.65074300
H	1.10083700	-1.79468700	0.71297300
C	3.81137500	0.36623000	0.39090800
H	3.37638000	0.74536100	-1.68962600
H	4.88734700	0.22717500	0.21332400
H	3.72428600	0.91286000	1.34331500
C	3.66480800	-1.68888900	1.83836400
H	3.19939300	-2.67084600	1.99251300
H	4.75071900	-1.83919400	1.78396700
H	3.45490700	-1.07476500	2.72324300
C	3.46481800	-1.94029300	-0.64077500
H	4.54792200	-2.10124900	-0.71487700
H	2.98842400	-2.92044500	-0.51611200
H	3.12681300	-1.53414700	-1.59984100
C	3.90501000	2.61452200	-0.78957900
H	4.98191500	2.50471000	-0.96661700
H	3.49319900	3.23079000	-1.59840800
H	3.77578200	3.16666800	0.15084900

Octisalate A

C	-5.16404400	0.59618800	1.04670200
C	-4.03428600	1.38837700	1.31327400
C	-2.79691100	0.99256300	0.82616000
C	-2.65739900	-0.18940100	0.07009800

C	-3.80628400	-0.98174100	-0.19133500
C	-5.05532000	-0.57282100	0.30536100
H	-6.13786000	0.89826300	1.42427400
H	-4.12736000	2.30068500	1.89488400
H	-1.91292500	1.58998000	1.02148400
H	-5.91977100	-1.19426900	0.09258500
O	-3.76253600	-2.12484200	-0.90357000
H	-2.82348500	-2.27265200	-1.17633300
C	-1.35300700	-0.62581100	-0.45650100
O	-1.19183000	-1.65826000	-1.11548300
O	-0.33485500	0.19592100	-0.16208500
C	0.98054900	-0.18797200	-0.64395900
H	0.93366400	-0.29595000	-1.73352200
H	1.22300300	-1.16462700	-0.21699500
C	1.97918100	0.89306000	-0.23132200
H	1.84845200	1.06695700	0.84804500
C	3.42011800	0.38593200	-0.47281700
H	3.48276700	-0.04946300	-1.48265900
H	4.10089000	1.24673500	-0.47840600
C	3.93723300	-0.62894200	0.55889500
H	3.27940100	-1.50891100	0.59618300
H	3.89523000	-0.17445000	1.56032900
C	5.37220900	-1.09735000	0.27766000
H	6.03757100	-0.22235500	0.23966800
H	5.41378800	-1.55600300	-0.72100800
C	5.89744400	-2.09334300	1.31835300
H	5.27218600	-2.99464200	1.35607200

H	6.92204400	-2.40875800	1.08774100
H	5.90304900	-1.65154900	2.32313400
C	1.69361900	2.21849600	-0.97169700
H	2.00797100	2.11327800	-2.02125200
H	0.60977000	2.38715200	-0.98981900
C	2.36856900	3.44816700	-0.35028100
H	3.46148300	3.36158400	-0.33584600
H	2.12063300	4.35473800	-0.91488600
H	2.03324200	3.59929600	0.68387700

Octisalate B

C	-5.35707700	-0.08787500	0.75719400
C	-5.10478400	-1.16943000	-0.10145800
C	-3.80931700	-1.39374900	-0.54331000
C	-2.74140900	-0.55996300	-0.15155100
C	-3.01118200	0.52703700	0.71745900
C	-4.32483400	0.74865600	1.16158000
H	-6.36701500	0.10139300	1.11241000
H	-5.91194400	-1.82370800	-0.41713400
H	-3.58178700	-2.22206800	-1.20679400
H	-4.50267100	1.58858200	1.82624300
O	-2.07260000	1.39501000	1.16921300
H	-1.20645900	1.14202400	0.79257600
C	-1.40273600	-0.90507900	-0.68969600
O	-1.16156900	-1.85615900	-1.40706300
O	-0.42118600	-0.03200500	-0.30025300
C	0.92913800	-0.31079400	-0.76517200

H	0.90801600	-0.37498900	-1.85811500
H	1.21807700	-1.28883900	-0.37250600
C	1.85293400	0.80903500	-0.28606400
H	1.68025700	0.94516600	0.79340200
C	3.32586700	0.38222900	-0.48851400
H	3.44306100	-0.02697900	-1.50437900
H	3.96078400	1.27660400	-0.45365800
C	3.85973100	-0.62767500	0.53952600
H	3.23920100	-1.53506300	0.54544100
H	3.77298700	-0.19285800	1.54677900
C	5.31986800	-1.03041900	0.28784700
H	5.94841600	-0.12788000	0.28021100
H	5.40558500	-1.47073900	-0.71622500
C	5.86021100	-2.02050900	1.32645000
H	5.27245100	-2.94743000	1.33428400
H	6.90251800	-2.28930100	1.11731600
H	5.82220800	-1.59530400	2.33763600
C	1.52872300	2.13921400	-1.00298300
H	1.90019300	2.08100200	-2.03715900
H	0.43958300	2.25255300	-1.07702200
C	2.10209100	3.38508700	-0.31554000
H	3.19577800	3.35574200	-0.24545400
H	1.83455100	4.29207000	-0.87007200
H	1.70571300	3.49065500	0.70242800

Octocrylene

C	3.47582500	-2.24737300	-0.91648000

C	4.54935900	-3.13549100	-0.85538200
C	5.60580100	-2.90234000	0.02947500
C	5.59178900	-1.76511700	0.84308600
C	4.53031500	-0.86448200	0.76818400
C	3.44723200	-1.10145900	-0.10048800
H	2.66540200	-2.43379600	-1.61377500
H	4.55861600	-4.01053400	-1.49958200
H	6.43789500	-3.59974300	0.08101600
H	6.41021300	-1.57676200	1.53290500
H	4.52814900	0.02130800	1.39646700
C	2.32187000	-0.13476800	-0.16378400
C	2.68868200	1.30569200	-0.13646800
C	3.66157000	1.80480300	-1.01926300
C	2.11990200	2.17641500	0.80794100
C	4.02304300	3.15150900	-0.98678800
H	4.11873500	1.13821400	-1.74517600
C	2.50030900	3.51697900	0.85632300
H	1.38887600	1.79478100	1.51583500
C	3.44597100	4.01092400	-0.04713000
H	4.75994400	3.52833300	-1.69121800
H	2.05764400	4.17588200	1.59875200
H	3.73645800	5.05781000	-0.01459800
C	1.01329200	-0.54848500	-0.19572200
C	0.64889800	-1.91377900	0.04717000
C	-0.13380700	0.37051200	-0.52207600
O	-0.05539600	1.35768500	-1.22537900
N	0.31508600	-3.01184500	0.24732400

O	-1.27656400	-0.07283000	0.03170300
C	-2.48653600	0.65878500	-0.29938500
H	-2.55048300	0.71267700	-1.39060700
H	-2.38912900	1.68265400	0.08099800
C	-3.69538800	-0.06647300	0.30012600
H	-3.53689100	-1.14339900	0.13772300
C	-3.86494600	0.18351700	1.81470000
H	-4.06327400	1.25601700	1.96990700
H	-4.76758300	-0.34147500	2.14934000
C	-2.69891600	-0.25588900	2.71160500
H	-2.44947500	-1.31153700	2.55291500
H	-1.79010500	0.32471100	2.52471200
H	-2.96630000	-0.12692300	3.76766000
C	-4.95417900	0.35547000	-0.49587900
H	-4.75023700	0.21365900	-1.56726200
H	-5.12636900	1.43490100	-0.35956400
C	-6.24171100	-0.40828700	-0.15088400
H	-6.54241100	-0.20277700	0.88512100
H	-6.04728000	-1.49037700	-0.20724400
C	-7.41134900	-0.05669300	-1.08191300
H	-7.59550500	1.02685200	-1.03829600
H	-7.12667000	-0.27641400	-2.12118100
C	-8.70323000	-0.80744500	-0.73774900
H	-9.03482800	-0.57986700	0.28357900
H	-9.51736500	-0.53554800	-1.42033400
H	-8.56072400	-1.89372200	-0.80527300

Bemotrizinol

C	-2.44043300	0.00469400	-0.09752400
C	-3.68905500	0.68756600	-0.13148100
C	-2.46571100	-1.40975300	-0.08586000
C	-4.89221900	-0.03578900	-0.14849000
C	-3.64348800	-2.12230200	-0.10058100
H	-1.51451800	-1.92878600	-0.06117200
C	-4.87326100	-1.42805100	-0.13124100
H	-5.81274400	0.53290500	-0.17766300
H	-3.65572500	-3.20674900	-0.08834000
O	-5.98262300	-2.20826200	-0.14462300
C	-1.16242600	0.71042600	-0.07318100
C	1.12061900	0.69335000	-0.06889900
C	-0.00608600	2.69811900	0.00764200
N	-0.02604500	-0.00155900	-0.09873600
N	-1.18811800	2.06907300	-0.02931800
N	1.16668700	2.05030500	-0.00538500
C	2.38840300	-0.03096200	-0.10115700
C	2.39309400	-1.44014000	-0.22441300
C	3.64669200	0.62895600	-0.01519700
C	3.56049100	-2.16871000	-0.26659800
H	1.43444600	-1.94174200	-0.28905900
C	4.83927000	-0.11066500	-0.05669100
H	5.76810000	0.44072100	0.01342300
O	5.89815900	-2.29180900	-0.23187100
C	0.00459600	4.17252300	0.06491400
C	1.16197500	4.89513300	-0.25568800

C	-1.14753400	4.88967600	0.44145600
C	1.18307600	6.28670800	-0.21067600
H	2.05462200	4.36275600	-0.56547300
C	-1.13719200	6.27263700	0.49926800
H	-2.04899700	4.34996400	0.70987300
C	0.02889500	6.98506400	0.17131500
H	2.09286500	6.81242800	-0.47557200
H	-2.01836700	6.83057700	0.79902600
O	-0.06414700	8.33747400	0.25508600
O	3.77183800	1.96127900	0.11625600
H	2.84639200	2.33145000	0.13830700
O	-3.79531500	2.02738100	-0.15823900
H	-2.86543200	2.38604400	-0.15009800
H	3.55693600	-3.24887900	-0.36431700
C	4.80008400	-1.49704900	-0.18190800
C	1.08223700	9.11863100	-0.05592400
H	1.40035000	8.96814100	-1.09535600
H	1.91831400	8.88952800	0.61688900
C	7.19641700	-1.69658300	-0.14694800
H	7.28107300	-1.13068500	0.79090000
H	7.32874800	-0.99071200	-0.97852900
C	8.24652800	-2.80796000	-0.22242700
H	8.07425900	-3.33112900	-1.17475000
C	-7.27239100	-1.58934300	-0.17186200
H	-7.38285200	-0.93070500	0.70043200
H	-7.35990900	-0.96915500	-1.07458900
C	-8.33845600	-2.68794500	-0.17794600

H	-8.13860500	-3.30681600	-1.06528100
C	9.65026700	-2.16983700	-0.28915800
H	9.84444300	-1.59588600	0.62828300
H	9.65916900	-1.43555300	-1.10816700
C	-9.72700000	-2.04060200	-0.36509700
H	-9.94641600	-1.37414200	0.48136900
H	-9.69119100	-1.39295400	-1.25349600
C	10.79813600	-3.16336900	-0.51522200
H	10.86576800	-3.85966800	0.33166500
H	10.57730800	-3.77943400	-1.39971300
C	-10.88253100	-3.03629000	-0.53604500
H	-10.63858300	-3.74148800	-1.34463700
H	-10.99481800	-3.64251500	0.37310400
C	12.15774400	-2.47708900	-0.70406900
H	12.10023800	-1.78827700	-1.55887500
H	12.37326200	-1.85263900	0.17473900
C	-12.22170100	-2.35438800	-0.84714700
H	-12.11932800	-1.75609500	-1.76366100
H	-12.46011100	-1.64169900	-0.04470900
C	13.30733300	-3.46618100	-0.92145700
H	13.13673600	-4.08267500	-1.81265500
H	14.26277800	-2.94610200	-1.05453700
H	13.41391900	-4.14485600	-0.06620300
C	-13.37899300	-3.34494000	-1.01029000
H	-14.31949000	-2.82821100	-1.23282100
H	-13.18491000	-4.05018600	-1.82782600
H	-13.53007100	-3.93263400	-0.09638200

H	0.77992400	10.15777400	0.08296800
C	-8.22786300	-3.61941300	1.05116900
H	-7.21348000	-4.03318200	1.06735500
H	-8.90111500	-4.47260200	0.90187000
C	-8.52910900	-2.97552700	2.41094300
H	-8.40855400	-3.71112900	3.21410300
H	-7.85021700	-2.14363900	2.63137300
H	-9.55474000	-2.59316300	2.46981100
C	8.07311800	-3.85568900	0.90125900
H	7.05327200	-4.25072800	0.83580200
H	8.73996300	-4.70160000	0.69327100
C	8.32742900	-3.35688400	2.32982700
H	7.65200400	-2.53896100	2.60610100
H	8.16342800	-4.16693900	3.04927600
H	9.35515900	-3.00134700	2.46742500

Molecular dynamics

Metodology

As a first step in the construction of the models studied in classical molecular dynamics, we considered the following number of atoms and molecules as available in Table S2.

Table S2: Summary of the simulation details.

Molecule types	N° of atoms total systems	N° of molecules total systems
Avobenzone-keto	720	16
Avobenzone-enol	720	16
Bemotrizinol	855	9
Octisalate A	640	16
Octocrylene	864	16
Homosalate B	656	16
Water	48750	16520

Posteriorly, individual simulation boxes with lengths Lx= 10.0 nm, Ly = 6.0 nm, and Lz= 2.0 nm to contain the solute molecules in the numbers presented in Table S1. These dimensions were the same for all boxes. An initial representation of these boxes can be seen in Figure S9. The same box dimensions were reproduced for all solute molecules.

Based on the model, we performed energy minimization using the force-field parameters available for each molecular system. Such parameters are included in the appendix to this supplementary material in files with the .top extension.

After minimization, the boxes containing the solute molecules are then placed within a larger box containing water as the solvent, with dimensions L_x= 10.0 nm, L_y= 10.0 nm, and L_z= 5.0 nm. This final box (the latter) was used to perform the subsequent molecular dynamics calculations; see Figure S10 for an example of the representation.

For the intermolecular potential model between molecules i and j, it was considered the sum of the interactions between each atomic pair of such molecules, which are given by a Lennard-Jones (LJ) term, plus the corresponding term for the electrostatic interactions between each pair of sites a and b belonging to molecules i and j, respectively:

Figure S9: Top and side view of the box containing Bemotrizinol molecules, with dimensions $L_x = 10.0$ nm, $L_y = 6.0$ nm, and $L_z = 2.0$ nm.

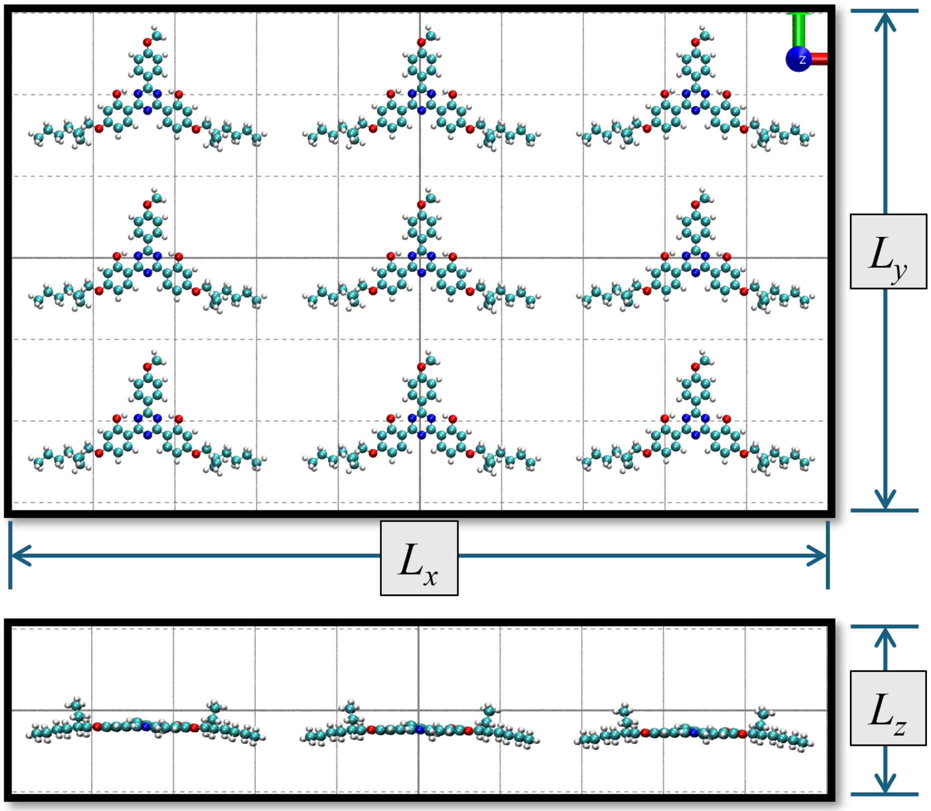

Figure S10: Model box contained Avobenzone(keto)/water molecules. Here L_x = 10.0 nm, L_y = 10.0 nm and L_z= 5.0 nm

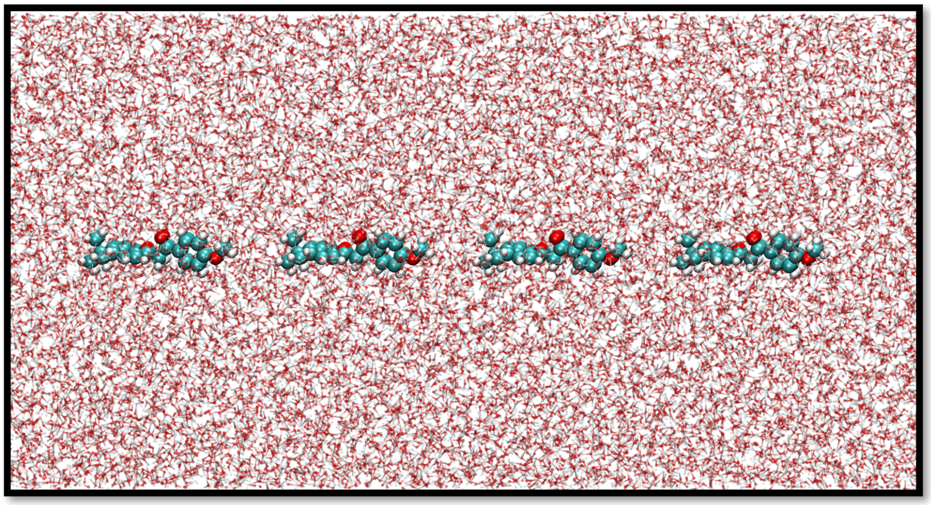

$$V_{ab} = \sum_{ab} \left\{ 4\varepsilon_{ab} \left[\left(\frac{\sigma_{ab}}{r_{ab}} \right)^{12} - \left(\frac{\sigma_{ab}}{r_{ab}} \right)^{6} \right] + \frac{q_a q_b}{4\pi\varepsilon_0 r_{ab}} \right\}; \qquad V_{ij} = \sum_{a,b \in i,j} V_{ab} \tag{S1}$$

where are the partial charges qa,b, and are the energy and diameter parameters of LJ of site aa and aa, and rab is the separation of sites a and b of distinct molecules. These same interactions are used to describe an intramolecular potential. To calculate the interaction between sites of different species, it is necessary to use some combination rule for the LJ coefficients. The Lorentz-Berthelot rules[1,2] are the most commonly used and given by:

$$\varepsilon_{ab} = \sqrt{\varepsilon_{aa}\, \varepsilon_{bb'}} \qquad \therefore \qquad \sigma_{ab} = \frac{\sigma_{aa} + \sigma_{bb}}{2}. \tag{S2}$$

For the bonded or intramolecular potentials, the molecular vibrations used in our models were:

$$V_{\text{intra}} = V_{\text{bond}} + V_{\text{angle}} + V_{\text{dihedral}} \tag{S3}$$

where represents the bonding potential V_{bond}, represents the angular vibration potential V_{angle}, and represents the vibration potential of the dihedral angles $V_{dihedral}$. The terms of

angular stretch and deformation are usually described by harmonic potentials in the form:

$$V_{\text{bond}} = \sum k_b (r - r_0)^2, \qquad V_{\text{angle}} = \sum k_\theta (\theta - \theta_0)^2 \tag{S4}$$

where r and r_0 correspond to the length and θ_0 the angle of equilibrium of bonding, and k_b, k_θ are the force constants. These parameters are obtained through spectroscopic measurements or quantum calculations. We may also have the potential for torsion,

$$V_{\text{dihedral}} = \sum k_\phi \left(1 + \cos\cos(M\phi - \Delta)\right) \tag{S5}$$

where k_ϕ is the torsion constant of the molecule, M is a factor of multiplicity, ϕ is the angle, and Δ is any phase.

Thus, starting from the functionals presented above, the expression for the determination of the potential on each of the molecules of the system under study assumes the general form:

$$V_{\text{tot}} = V_{ab} + V_{\text{bond}} + V_{\text{angle}} + V_{\text{dihedral}} \tag{S6}$$

The coefficients required to model the total potential in S.6 are then described according to the topology file in LAMMPS format.[3]

Adittional Results

We present the radial distribution function (RDF) profiles for site-to-site distances within the same molecule, cases shown in Figures S11 to S13, and between the oxygen site of the water molecule O_w) and sites on the different solute molecules, see Figures S14 to S16. The sites for which the distances were estimated are indicated by arrows in the images adjacent to the graphs.

Figure S11: Radial distribution function (RDF) of the intermolecular structures in: (a) Avobenzone(keto)/water, (b) Avobenzone(enol)/water. O_d – oxygen binding by double bond; O_bc – oxygen connected in benzene;

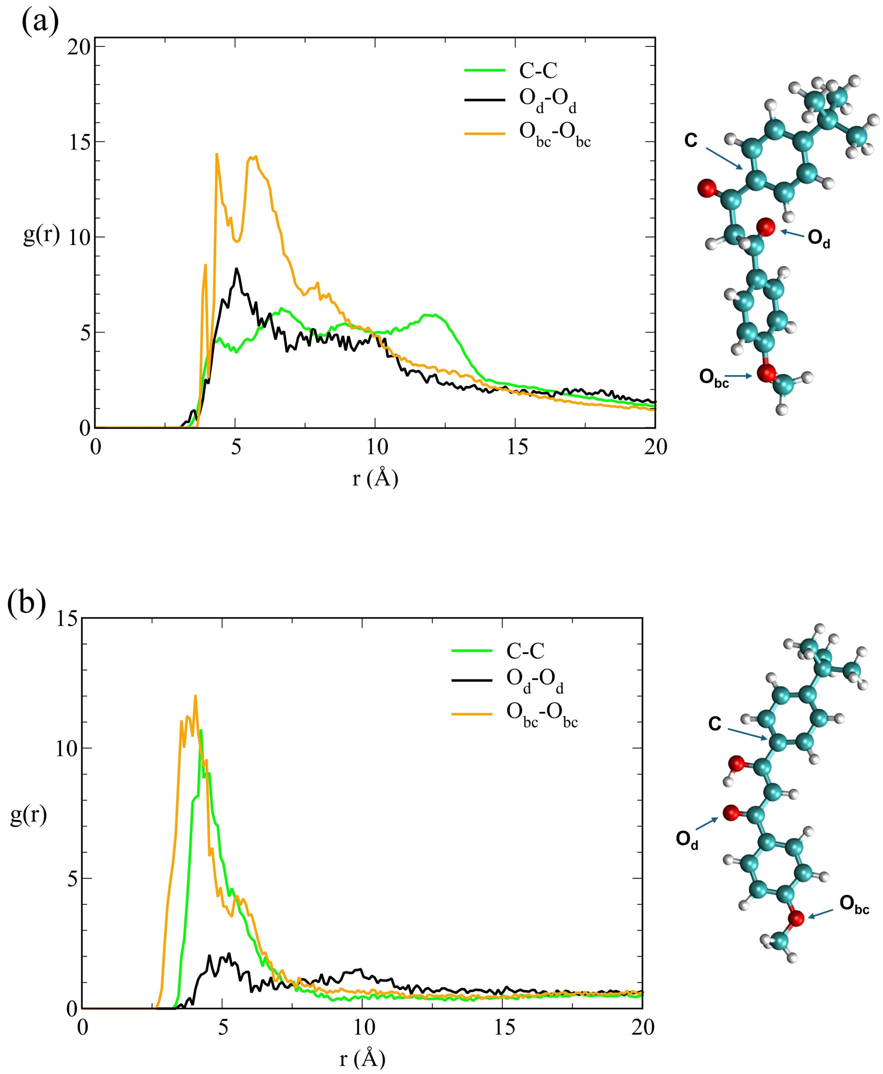

Figure S12: Radial distribution function (RDF) of the intermolecular structures in: (a) Bemotrizinol/water, (b) Octisalate A/water.

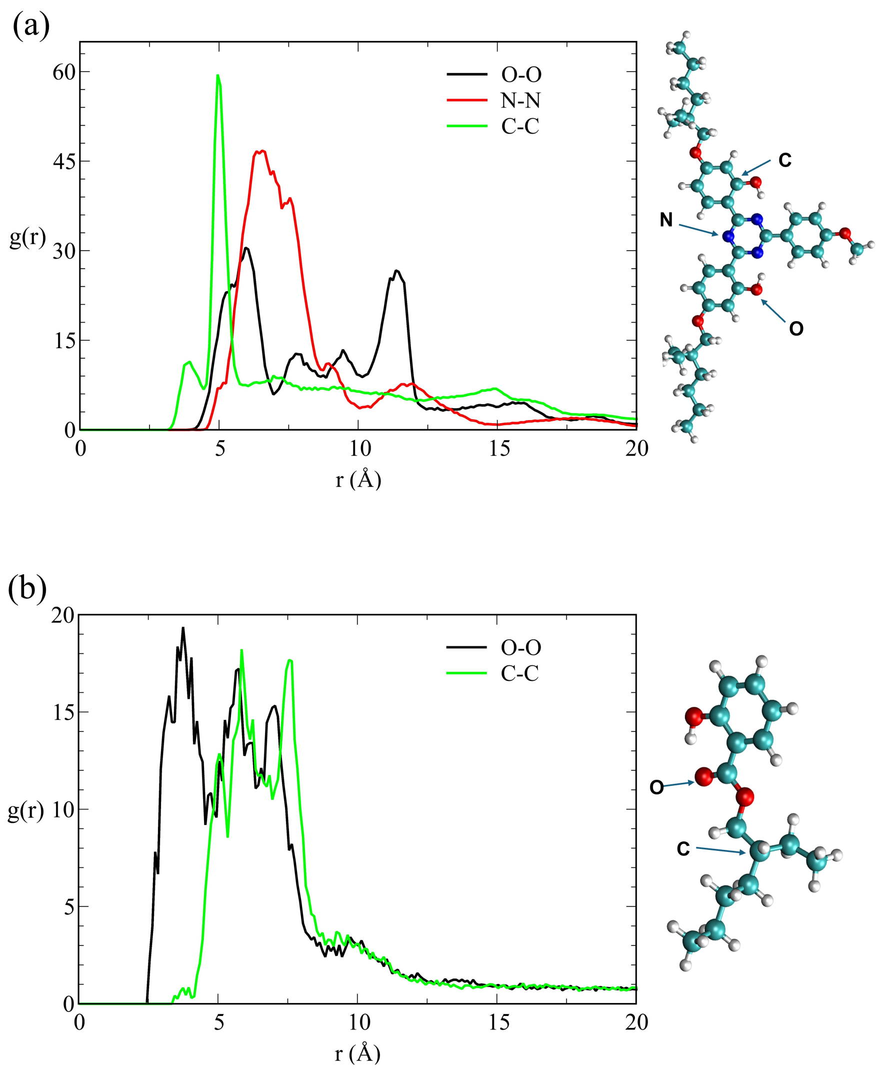

According to the results obtained from the density profiles, we assign an aggregation scale for the solute molecules in the order from most aggregated to least aggregated as follows: Octocrylene > Bemotrizinol > Avobenzone-keto > Octisalate A > Avobenzone-enol > Homosalate B. It is therefore noted that this order can be applied when analyzing the intensities of the g(r) (radial distribution function) of different solute types.

Figure S13: Radial distribution function (RDF) of the intermolecular structures in: (a) Octocrylene/water, (b) Homosalate/water.

(a)

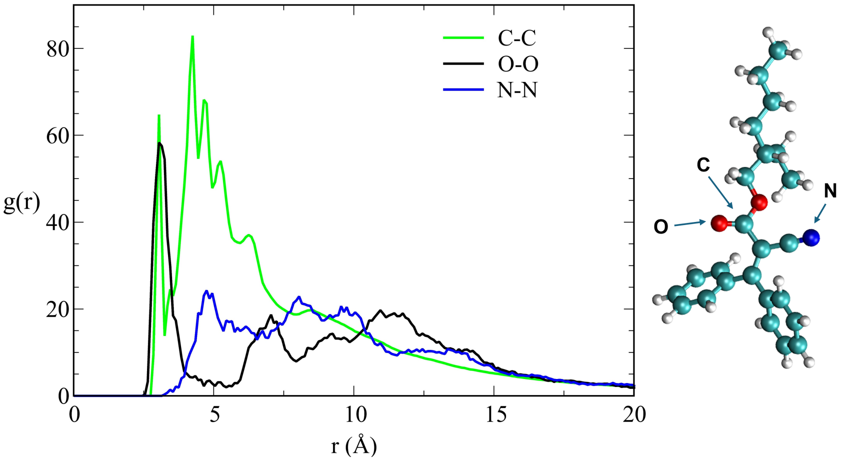

(b)

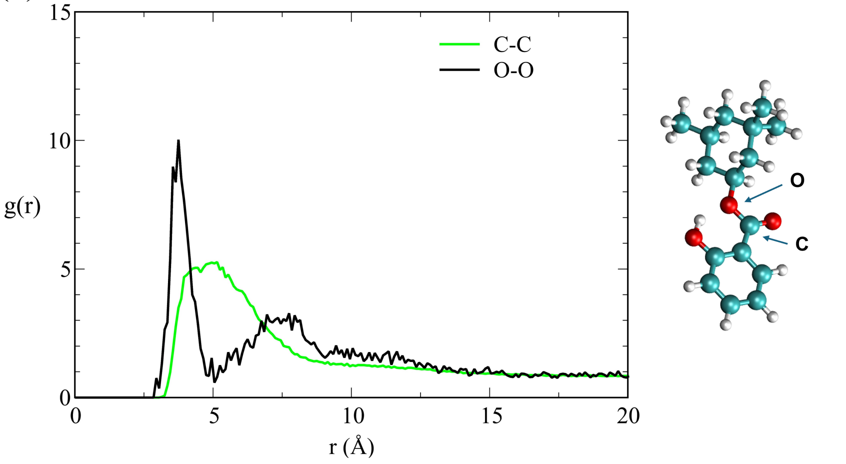

Figure S14: Radial distribution function (RDF) of the intermolecular structures in: (a) Avobenzone(keto)/water, (b) Avobenzone(enol)/water. O_d – oxygen binding by double bond; O_bc – oxigen conected in benzene;

(a)

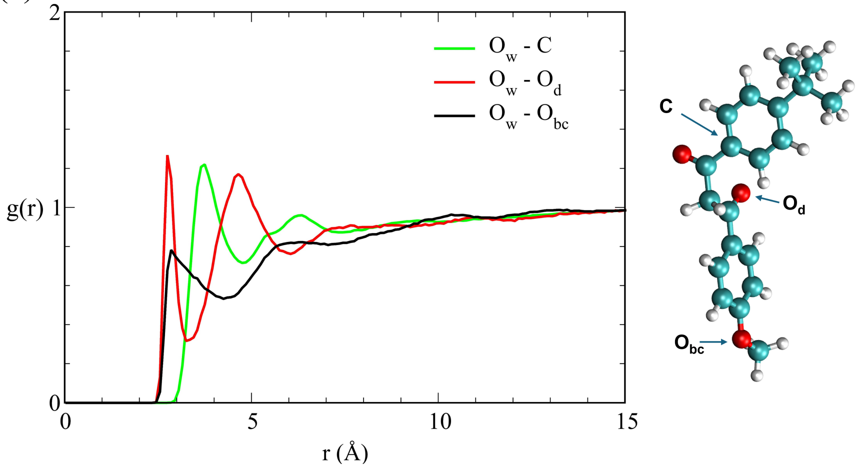

(b)

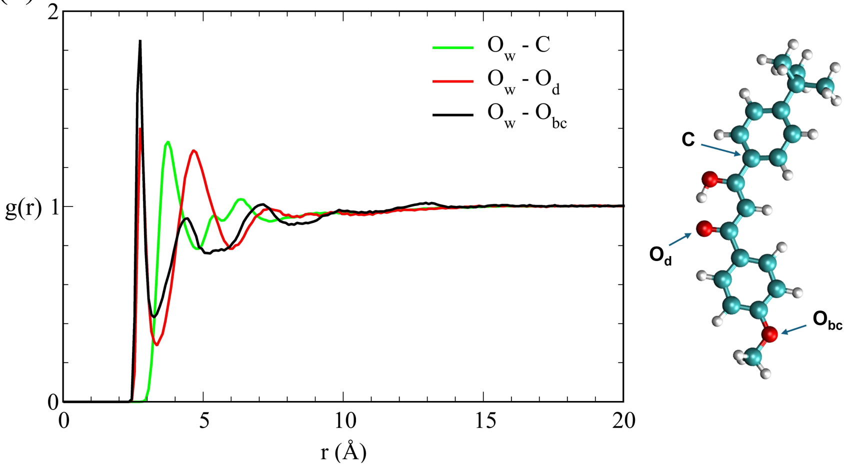

Figure S15: Radial distribution function (RDF) of the intermolecular structures in: (a) Bemotrizinol/water, (b) Octisalate A/water.

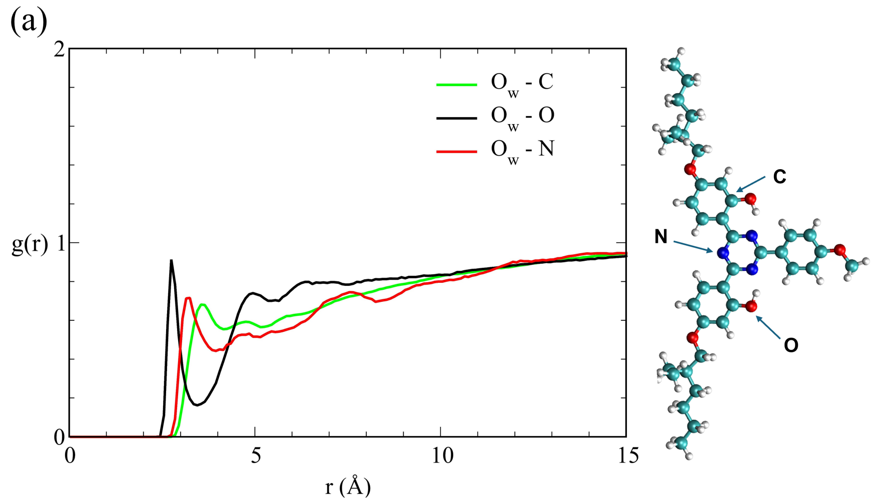

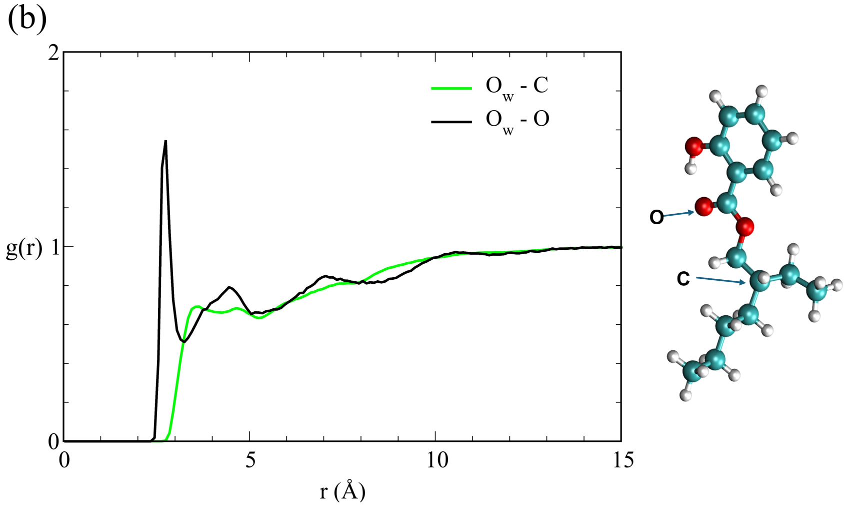

Figure S16: Radial distribution function (RDF) of the intermolecular structures for (a) Octocrylene/water, (b) Homosalate B/water.

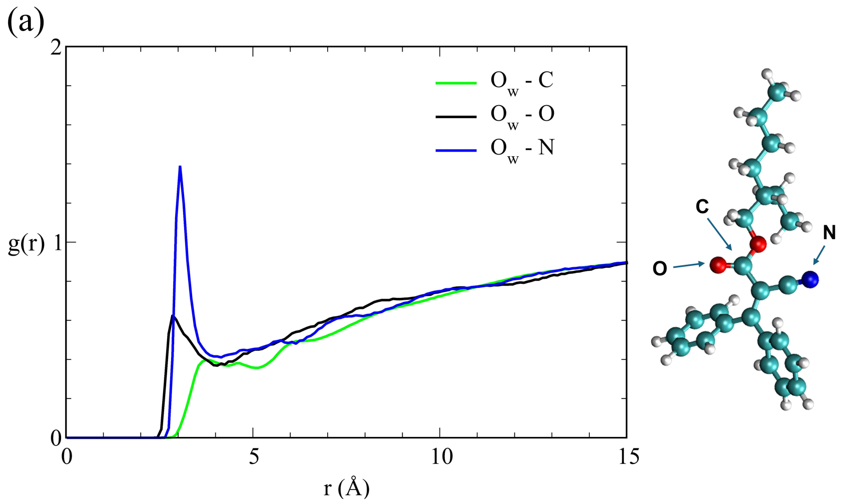

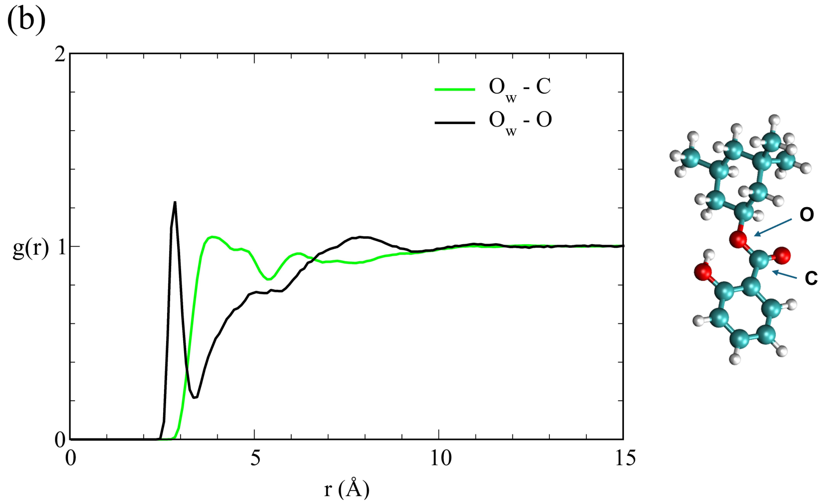

Figure S17: (a) The estimated radius of gyration, determined over the final 20 ns interval of the molecular dynamics simulations, was recorded. (b) zoom according to (a).

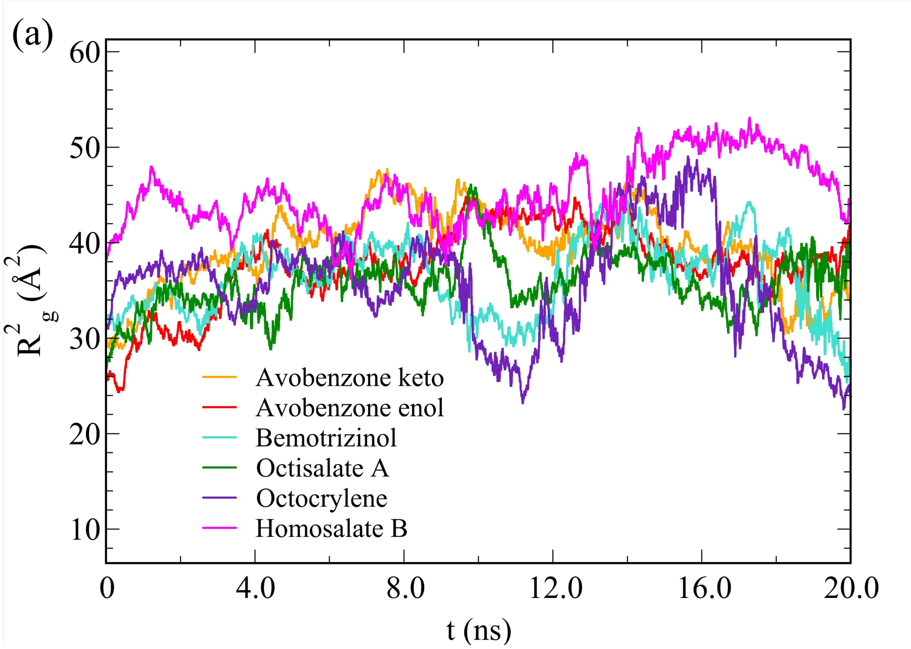

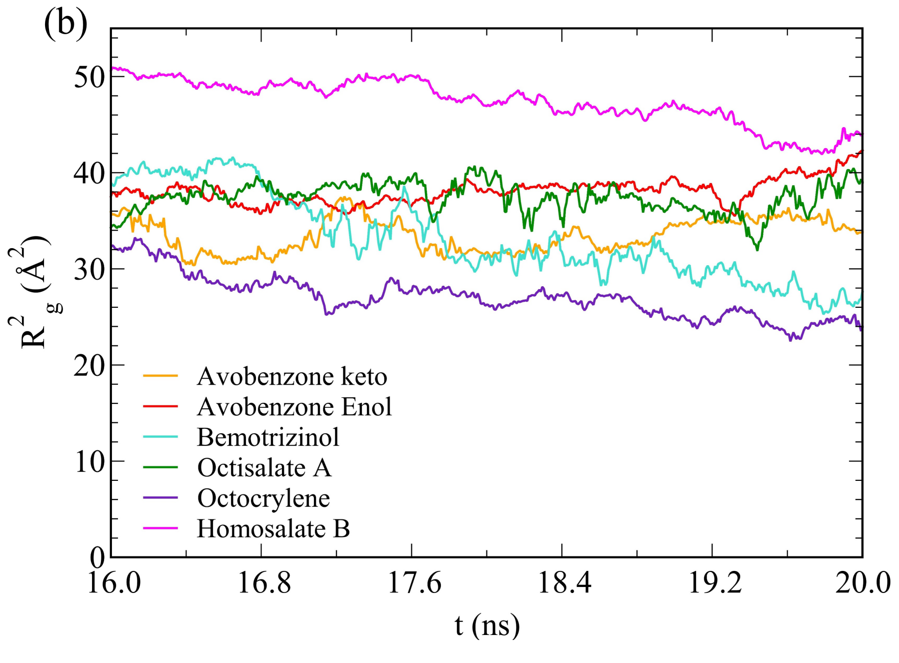

The same analysis of solute aggregation is supported by the radius of gyration (Fig. S9) of these aggregates in the solvent.

References

(1) Lorentz, H. A. On the Application of the Theorem of the Virial in the Kinetic Theory of Gases. *Annalen der Physik* **1881**, *12*, 127–136.

(2) Berthelot, D. Sur le mélange des gazes. *Comptes rendus hebdomadaires des séances de l'Académie des Sciences* **1898**, *126*, 1703–1855.

(3) Thompson, A. P.; Aktulga, H. M.; Berger, R.; Bolintineanu, D. S.; Brown, W. M.; Crozier, P. S.; in 't Veld, P. J.; Kohlmeyer, A.; Moore, S. G.; Nguyen, T. D.; Shan, R.; Stevens, M. J.; Tranchida, J.; Trott, C.; Plimpton, S. J. LAMMPS – a flexible simulation tool for particle-based materials modeling at the atomic, meso, and continuum scales. *Computer Physics Communications* **2022**, *271*, 108171.
